# Traditional Masculinity and Femininity: Validation of a New Scale Assessing Gender Roles

**DOI:** 10.3389/fpsyg.2016.00956

**Published:** 2016-07-05

**Authors:** Sven Kachel, Melanie C. Steffens, Claudia Niedlich

**Affiliations:** Department of Social and Economic Psychology, University of Koblenz and LandauLandau, Germany

**Keywords:** gender stereotypes, gender roles, gender-role self-concept, femininity, masculinity, actual and perceived sexual orientation, scale construction, voice pitch characteristics

## Abstract

Gender stereotype theory suggests that men are generally perceived as more masculine than women, whereas women are generally perceived as more feminine than men. Several scales have been developed to measure fundamental aspects of gender stereotypes (e.g., agency and communion, competence and warmth, or instrumentality and expressivity). Although omitted in later version, Bem's original Sex Role Inventory included the items “masculine” and “feminine” in addition to more specific gender-stereotypical attributes. We argue that it is useful to be able to measure these two core concepts in a reliable, valid, and parsimonious way. We introduce a new and brief scale, the Traditional Masculinity-Femininity (TMF) scale, designed to assess central facets of self-ascribed masculinity-femininity. Studies 1–2 used known-groups approaches (participants differing in gender and sexual orientation) to validate the scale and provide evidence of its convergent validity. As expected the TMF reliably measured a one-dimensional masculinity-femininity construct. Moreover, the TMF correlated moderately with other gender-related measures. Demonstrating incremental validity, the TMF predicted gender and sexual orientation in a superior way than established adjective-based measures. Furthermore, the TMF was connected to criterion characteristics, such as judgments as straight by laypersons for the whole sample, voice pitch characteristics for the female subsample, and contact to gay men for the male subsample, and outperformed other gender-related scales. Taken together, as long as gender differences continue to exist, we suggest that the TMF provides a valuable methodological addition for research into gender stereotypes.

## Introduction

Every time a group of people is addressed as “Ladies and Gentlemen!” the pervasiveness of gender over all other social categories is demonstrated. Gender is also one of the first social categories that children learn in today's societies, and thus knowledge of gender stereotypes is evident from early childhood on (for a recent review, see Steffens and Viladot, 2015) and into adulthood, with both adolescents and college students construing their self-concepts in line with the gender stereotypes they have internalized (e.g., Nosek et al., 2002; Steffens et al., 2010). Since the 1970s, following Bem's (1974) pioneering work, many scales have been designed, developed, and widely used for measuring traits traditionally considered as typically male vs. typically female (Constantinople, 1973). In recent years, such measures have often failed to find between-gender differences in self-ascriptions of gender stereotypical traits (e.g., Sczesny et al., 2004), which is presumably due to changes in gender roles across the decades (e.g., Diekman and Eagly, 2000; Wilde and Diekman, 2005; Ebert et al., 2014). Still, gender differences in self-ascriptions do continue to exist, and there are attempts to measure different aspects of masculinity and femininity, including, for example, everyday behavior such as housework (Athenstaedt, 2003). In the present paper, we argue that a scale that reliably and validly measures differences in an individual's underlying conceptualization of his or her own masculinity-femininity would be valuable for gender research. To date, these constructs can only be measured using two items, “masculine” and “feminine,” which is somewhat limited given that established standards of psychological assessment typically recommend using a larger number of items (e.g., Bühner, 2010). In the present article, we introduce a new, extended, but still parsimonious scale, the Traditional Masculinity-Femininity Scale, TMF, to fill this gap. Using a known-groups approach, we present two studies testing this measure's reliability as well as its incremental and criterion validity, and we provide evidence for its convergent validity.

We define “traditional masculinity” and “traditional femininity” as relatively enduring characteristics encompassing traits, appearances, interests, and behaviors that have traditionally been considered relatively more typical of women and men, respectively (adapting the definitions provided by Constantinople, 1973). It is important to note that the focus of the present paper is on gender-related self-assessment. Complementary research has investigated many different aspects of gender, for example, gender-role norms (e.g., Athenstaedt, 2000; Thompson and Bennet, 2015; Klocke and Lamberty, unpublished manuscript).

In a seminal study on masculinity and femininity, Deaux and Lewis (1984) investigated the perceived relationship between gender and gender-related components, such as role behaviors (e.g., head of household vs. takes care of children), traits, occupations, and physical characteristics (e.g., tall, broad-shouldered vs. soft voice, graceful). The researchers showed that these components were interdependent, impacting on one another, as well as on perceived gender and sexual orientation. In other words, participants readily generalized from one component to the others. In addition, physical appearance played a particularly large role. Such findings indicate that gender stereotypes may be based on some sort of “core” masculinity and femininity. Similarly, individuals may use such “core” masculinity and femininity in their self-construal.

The first attempts to gauge masculinity and femininity placed these constructs on a bipolar spectrum and involved measuring simple collections of personality traits on which women and men differed on average (for a review, see Constantinople, 1973). By contrast, Bem's pioneering Sex Role Inventory (BSRI; Bem, 1974) used gender-stereotypical traits to independently measure masculinity and femininity (e.g., masculine items such as competitive and dominant, and feminine items such as affectionate and gentle). She pointed out that women/men who score high on both scales were called androgynous. Importantly, “masculine” and “feminine” were included as items in these original scales, but were excluded from the revised version (Bem, 1979) because of problematic loadings on the factors on which the masculine and feminine traits loaded, respectively. Exploratory factor analyses showed an instable factor structure but often converged on three-factor solutions: Masculine traits on one factor, feminine traits on a second factor, and masculine-feminine along with participant gender on a third factor (e.g., Niedlich et al., 2015, see review by Choi and Fuqua, 2003). It has thus been suggested that the two independent masculinity and femininity trait dimensions are complemented by one bipolar masculinity-femininity dimension (see Constantinople, 1973; Spence et al., 1975; Bem, 1979) that reflects gender identity instead of gender-role related aspects (e.g., Bem, 1979; Spence and Buckner, 2000). As Choi and Fuqua (2003) suggest, inventories such as the BSRI “may not capture the complex and multidimensional nature of masculinity/femininity.” Instead, “masculinity and femininity could be two higher order constructs, with each having its own subconstructs” (p. 873). Similar to other scales (e.g., Personal Attributes Questionnaire, PAQ, by Spence et al., 1975), the BSRI appears to tap more specific constructs, often referred to as instrumentality/agency and expressivity/communion (e.g., Fiske et al., 2002; Abele and Wojciszke, 2007), rather than masculinity and femininity in general. For the present purposes it is important to note that if masculinity and femininity are directly measured they should load on one bipolar masculinity-femininity dimension.

Another limit to the practical use of these established scales pertains to the generally small magnitude of gender differences found on these two dimensions (e.g., Deaux, 1984). In other words, women and men appear rather similar on “masculinity” and “femininity.” More recently, gender differences have not emerged at all between graduates with the same major (see Abele, 2000). In short, scales that have been developed to assess aspects of masculinity and femininity have recently failed to find gender differences (see also Sczesny et al., 2004; Evers and Sieverding, 2014). This could indicate that gender differences in masculinity and femininity are a thing of the past (Alvesson, 1998). However, it could also mean that the scales do not tap the most relevant aspects of the constructs on which gender differences continue to exist. For example, gender roles have changed over the last decades, particularly women's roles, so that today's women possess more of the traits traditionally considered as masculine (e.g., Diekman and Eagly, 2000; Spence and Buckner, 2000; Wilde and Diekman, 2005; Ebert et al., 2014). According to these findings, instrumental traits have become more socially desirable for women and expressive traits have become more socially desirable for men (Swazina et al., 2004).

In order to overcome limitations of the discussed scales, there have been attempts to measure other aspects of masculinity and femininity to account for the multiple dimensions they are reflected in, such as physical appearance, behaviors, attitudes, and interests (e.g., Spence and Buckner, 2000; Blashill and Powlishta, 2009). For example, Athenstaedt (2003) observed considerable gender differences in everyday behavior such as “putting flowers on the desk” (feminine) and “putting the meat on the barbeque” (masculine), strongly suggesting the continued importance of gender differences. Complementing these existing approaches, we suggest directly assessing the presumed higher-order constructs, namely masculinity and femininity. However, instead of using only these two items, we constructed a scale that can be tested empirically with regard to its reliability and validity.

### Scale construction

We introduce the TMF scale, an instrument for measuring gender-role self-concept. Appendix A1 in Supplementary Material shows all items, both English translations and original German wordings. Each item initially included in scale construction was selected based on theoretical considerations, as outlined in the following. We argue that we can measure the “core” of masculinity/femininity by referring to three central aspects, identified by Constantinople (1973), that we summarize using the term gender-role self-concept: Namely, gender-role adoption, gender-role preference, and gender-role identity. Constantinople (1973) defines *gender-role adoption* as the actual manifestation (i.e., how masculine-feminine a person considers her- or himself) and *gender-role preference* as the desired degree of masculinity-femininity (i.e., how masculine-feminine a person ideally would like to be). According to Kagan (1964), *gender-role identity* refers to a comparison of gender-related social norms and the gender-related characteristics of the individual (e.g., how a person actually looks compared to expected gender-typical appearances according to societal norms). Hence, for gender-role identity social comparisons as well as references to different gender-related aspects are emphasized (e.g., looks, behaviors etc.), whereas gender-role adoption and preference are based on non-relative, absolute statements. Following the former approach, we use TMF as a reference point. Based on dimensions identified as important in previous research, the TMF encompasses gender-role identity with regard to physical appearance, behavior, interests, and attitudes and beliefs (e.g., Deaux and Lewis, 1984; Athenstaedt, 2003). As mentioned, *physical appearance* was shown to play a particularly large role in implicating other components of gender stereotypes (Deaux and Lewis, 1984). Athenstaedt (2003) advocated the inclusion of gender-stereotypical *behaviors* in addition to traits, so this domain was included in the TMF as well. Lippa (2008) found that gender-related *interests* were highly relevant in discriminating women and men as well as lesbians/gay men from straight people. Additionally, his study showed that instrumental and expressive traits were outperformed by these gender-related interests in predicting participants' gender. Consequently, we included gender-related interests in the TMF (instead of gender-related traits). Finally, regarding *attitudes and beliefs*, gender differences have often been found, for example, with regard to attitudes toward minority groups (e.g., Sidanius et al., 1994; Kite and Whitley, 1996). We therefore also included self-assessment of attitudes and beliefs in the TMF.

One advantage of the TMF is that each of the mentioned scale dimensions is measured on a global level and not by various specific indicator items. Different from the instruments described above, which infer masculinity-femininity from the degree of affirmation of specific traits and behaviors, the TMF aims to directly assess masculinity-femininity. For example, “Traditionally, my behavior would be considered as…” 1 (*not at all masculine*) to 7 (*very masculine*). We consider it an asset of the scale that it is thus independent of specific stereotype content regarding masculinity and femininity that depend on culture and time (e.g., intelligent and ambitious as masculine, childlike and shy as feminine, see BSRI; in the General Discussion we discuss how far this global conception can also be considered a limitation). The TMF consists of six items only: One for gender-role adoption (“I consider myself as…”), one for gender-role preference (“Ideally, I would like to be…”), and four for gender-role identity (“Traditionally, my 1. interests, 2. attitudes and beliefs, 3. behavior, and 4. outer appearance would be considered as…”) in order to measure an individual's gender-role self-concept in a parsimonious way. All of them have high face validity. Each item is to be independently rated in terms of femininity and masculinity. A 7-point-scale is used to gauge the extent to which the participant feels feminine or masculine, how feminine or masculine she or he ideally would like to be, and how feminine and masculine her or his appearance, interests, attitudes, and behavior would traditionally be seen. Construct validity is tested in the studies described below. The TMF was used with masculinity and femininity as two unipolar dimensions (Study 1: 1, *not at all masculine*, to 7, *very masculine*, and 1, *not at all feminine*, to 7, *very feminine*) vs. one bipolar dimension (pilot study, Study 2; 1, *very masculine*, to 7, *very feminine*) in order to check for dimensionality.

### Overview of the present research

We validated the TMF in various ways. First, we conducted an item analysis and a factor analysis. As suggested by findings reported by Bem (1979), Constantinople (1973), and Spence et al. (1975; see Introductory Section), the TMF's items should load on one factor and tap a one-dimensional masculinity-femininity construct. Hence, we expected the TMF to measure a one-dimensional gender-role self-concept (Hypothesis 1).

#### Validation by using the known-groups approach

Based on the idea that gender differences are not a thing of the past, as indicated in the introduction, a valid masculinity and femininity scale should show these gender differences. Therefore, we expected men and women to differ considerably on self-ascriptions on the TMF, with men being more masculine and less feminine than women (Hypothesis 2).

Moreover, a valid masculinity and femininity scale should show differences between people differing in sexual orientation. The essence of gender stereotypes of straight women and men is that they conform to traditional gender roles (e.g., Kite and Deaux, 1987; Kite and Whitley, 1996; Madon, 1997; Blashill and Powlishta, 2009). Lay people expect straight women to be more feminine and less masculine than lesbians, and straight men to be more masculine and less feminine than gay men. Similarly, straight women's and men's self-ascriptions are, on average, more gender-typed than those of lesbians and gay men (see meta-analysis by Lippa, 2005). Bisexual women were found to score on masculinity-femininity in between lesbians and straight women (Lippa, 2005). Therefore, we used the known-groups approach as an established method for testing a scale's validity (e.g., Howitt and Cramer, 2008). We expected lesbians' self-ascriptions on the TMF to be less feminine and more masculine compared to straight women (Hypothesis 3a). Bisexual women should score in between (Hypothesis 3b). Additionally, we expected straight men's self-ascriptions to be more masculine and less feminine compared to gay men (Hypothesis 3c).

Because straight women and men conform to gender roles more than lesbians and gay men, comparing lesbians and gay men constituted a stricter test of the TMF. Consistent with Hypothesis 2 and gender self-stereotyping but contradictory to implicit gender inversion theory (Kite and Deaux, 1987; which we turn to in General Discussion), we hypothesized lesbians to be more feminine and less masculine than gay men (Hypothesis 4).

The idea that differences in “core” masculinity and femininity underlie differences in lesbians' and gay men's vs. straight women and men's self-ascriptions in gender typicality can formally be conceived as masculinity-femininity mediating the relationship between sexual orientation and responses on scales such as the BSRI (Hypothesis 5).

#### Validation by implicit and explicit gender-related measures

A common critique of self-report measures is that they could reflect differences in social desirability more than “true” underlying differences in traits. Using implicit measures relying on response-time differences, such as an Implicit Association Test (IAT), may minimize this problem (Greenwald et al., 1998). Implicit measures are assumed to assess the impulsive system: Habitual, repeated, long-term associations between concepts (Strack and Deutsch, 2004), including self-related concepts (e.g., Steffens and Schulze-Koenig, 2006). We expected lesbians to describe themselves more masculine and less feminine than straight women (Hypothesis 6).

Adults' masculinity-femininity is related to (recalled) gender conformity during adolescence (e.g., Safir et al., 2003) and childhood (e.g., Lippa, 2008). Thus, gender-role instruments for assessing current traits and behaviors as well as recalled gender-typical behaviors, preferences, and interests during childhood were also suitable for testing convergent validity. We assumed all these characteristics to show moderate correlations with the TMF (Hypothesis 7).

Additionally, we expected the TMF to predict sexual orientation within one gender group better than other gender-related scales. We assumed the TMF to outperform other gender-related scales when predicting sexual orientation of women and men (Hypothesis 8).

#### Hypotheses based on criterion validity

As indicated above, lay people use gender-typicality as an indicator for judging someone's sexual orientation (Rieger et al., 2010; Valentova et al., 2011). People self-reporting gender-typical characteristics are likely to be perceived as straight, whereas people who do not display such characteristics are more likely to be perceived as lesbian or gay on pictures, videos, and speech recordings. Hence, targets who are perceived as straight could be those who self-describe as gender-typical in masculinity-femininity ratings (Hypothesis 9).

Additionally, there is some evidence that voice pitch characteristics, also called fundamental frequency features, of lesbians and gay men are shifted toward what is typical for straight women and men. Generally, compared to straight women, straight men show voice pitches that are lower on average, in variability, and in range (e.g., Pierrehumbert et al., 2004; Munson and Babel, 2007). Average voice pitch has been found to be lower in straight compared to gay men (Baeck et al., 2011) and higher in straight women compared lesbians (Camp, 2009). Hence, we assumed gender-typical masculinity-femininity self-ratings to be reflected in gender-typical patterns of voice pitch characteristics (Hypothesis 10).

Furthermore, contact frequency of straight women and men with lesbians and gay men is linked to attitudes toward them (e.g., Swank and Raiz, 2010): A lower contact frequency is connected to more negative beliefs about lesbians and gay men. One belief about lesbians and gay men is that they transgress gender roles, on average (e.g., Kite and Whitley, 1996). It thus seems plausible that people who are more gender-typical themselves are those who have less contact to lesbians and gay men and hold more negative beliefs. Hence, we assumed gender-typical masculinity-femininity self-ratings to be connected to more current contact with straight women and men and less current contact with lesbians and gay men (Hypothesis 11).

#### Hypotheses concerning test-retest reliability and predictive validity

Finally, the TMF was expected to show at least moderate test-retest reliabilities given that people were re-invited after a 1-years period (Hypothesis 12). From a scale validation perspective, it is desirable to present analyses in which the predictor is truly assessed before the criterion. Therefore, we expected at least moderate predictive validity for other gender-related features at second measurement (Hypothesis 13).

## Pilot study

The pilot study had two aims. First, we tested the factor structure of the scale's version that contained six bipolar items. We assumed the TMF items to load on one factor (Hypothesis 1). Additionally, we wanted to determine the appropriateness of every single item by using an item analysis. Second, we assessed the scale's validity using a known-groups approach (Hypothesis 2).

### Methods

At the end of an online survey that had a different purpose, participants filled in the 6-item version of the TMF (see Appendix in Supplementary Material) and indicated their gender (response options: male, female, both, none, no response). Overall 319 participants finished the study. Thirteen of them were excluded from further analysis because they described themselves as both male and female or neither or they did not disclose their gender. Data from 188 women and 118 men were used for analysis. Their age ranged from 18 to 41 (*M* = 23.6, *SD* = 3.1). They were students of different majors from different German universities (specifically, in Thuringia). Participants received no compensation for participation. Approval for all studies reported in this paper was obtained by the board of ethics (= human subjects committee) of the School of Humanities and Social Sciences at the Friedrich-Schiller-University of Jena. All studies were carried out in accordance with its recommendations, with written informed consent obtained from all participants in accordance with the Declaration of Helsinki.

### Results

In order to check for one-dimensionality of the TMF, an exploratory principal axis factoring (PAF) was conducted. Sample adequacy was confirmed by a Kaiser-Meyer-Olkin (KMO) criterion of 0.87. All items were suitable for factor analysis as indicated by item-specific KMO values >0.79 and moderate to high commonalities (0.57–0.88). According to a graphical scree-plot analysis, a one-factor solution was confirmed. There was a steep decline of explained variance from factor one (77%) to factor two (10%). Each of the six items was represented well by the factor (factor loadings ranged from 0.75 to 0.94).

Reliability of the TMF was high (Cronbach's α = 0.94). As indicated by the coefficients in Table 1, no items needed to be deleted to improve reliability. Item-specific homogeneity was high and ranged from 0.66 to 0.72 (see Table 1). Corrected item-total correlations ranged from 0.72 to 0.91, suggesting that each item represented the scale well. Moreover, item means ranged from 0.51 to 0.59. Accordingly, every item received almost equal masculinity and femininity ratings, indicating that averaged across the sample containing women and men, items received “androgynous” responses, as one would expect. When computing item “difficulties” separately for each gender group, findings pointed in the expected directions: “Difficulties” ranged from 0.18 to 0.35 for the male sample, indicating “masculine” responses, and from 0.60 to 0.85 for the female sample, indicating “feminine” responses.

**Table 1:** **Item Characteristics of the TMF in the Pilot Study for the Whole Sample (left-hand values, ***n*** = 306) and Separately for Men (middle values, ***n*** = 118) and Women (right-hand values, ***n*** = 188)**.

		**Corrected item-total correlation**	**Cronbach's α if item is deleted**	**Item means**	**Item homogeneity**	**Factor loading**
1.	I consider myself as …	0.91, 0.67, 0.63	0.91, 0.79, 0.76	0.56, 0.23, 0.77	0.78, 0.51, 0.46	0.94, 0.80, 0.76
2.	Ideally, I would like to be…	0.87, 0.51, 0.56	0.92, 0.81, 0.77	0.55, 0.20, 0.76	0.76, 0.42, 0.42	0.91, 0.67, 0.74
3.	Traditionally, my interests would be considered as…	0.77, 0.65, 0.56	0.93, 0.77, 0.77	0.51, 0.30, 0.63	0.69, 0.45, 0.41	0.84, 0.76, 0.71
4.	Traditionally, my attitudes and beliefs would be considered as…	0.72, 0.61, 0.67	0.94, 0.80, 0.74	0.51, 0.35, 0.60	0.66, 0.45, 0.45	0.81, 0.74, 0.80
5.	Traditionally, my behavior would be considered as…	0.85, 0.73, 0.67	0.93, 0.77, 0.74	0.52, 0.30, 0.66	0.75, 0.49, 0.45	0.90, 0.83, 0.79
6.	Traditionally, my outer appearance would be considered as…	0.83, 0.45, 0.26	0.93, 0.82, 0.82	0.59, 0.18, 0.85	0.72, 0.35, 0.21	0.88, 0.61, 0.39

Scale ranged from 1—“very masculine” to 7—“very feminine.”

**Figure 1:**
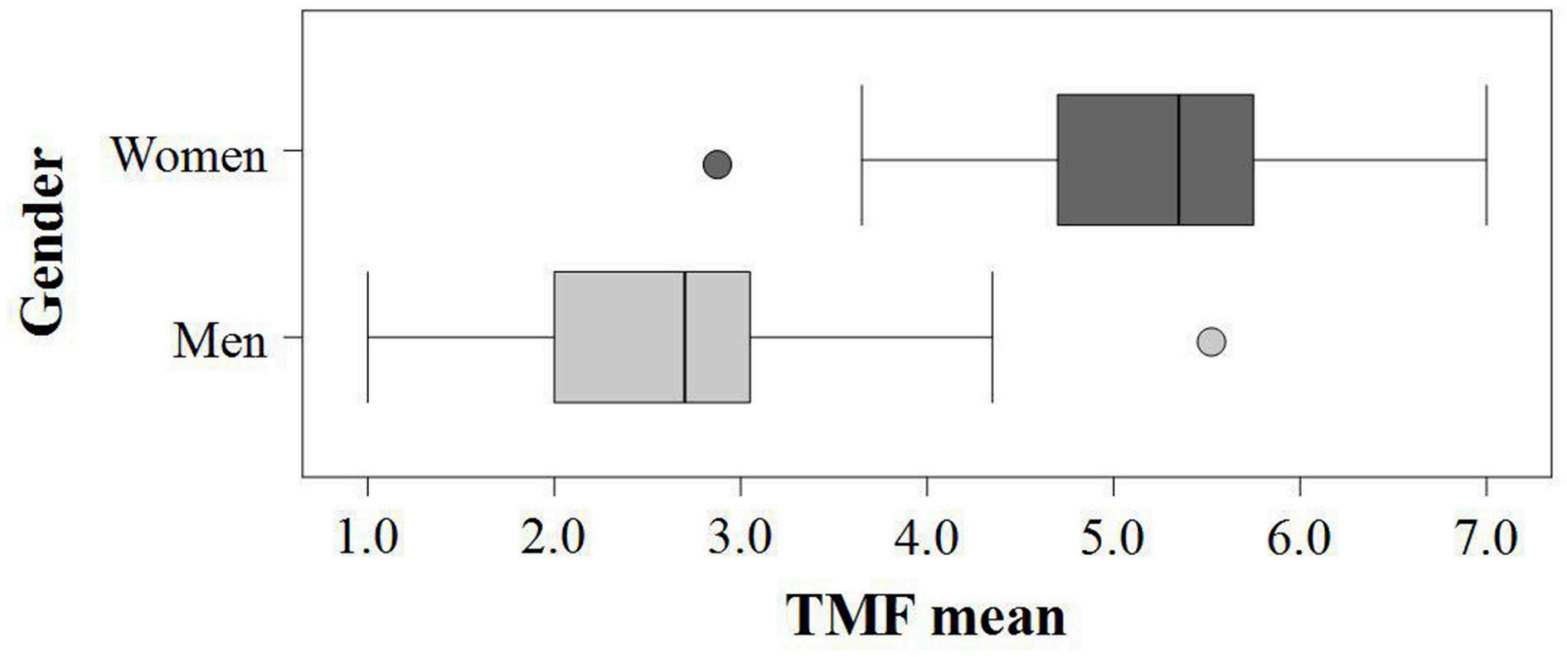
**Distribution of the TMF scores separately for men (***n*** = 118) and women (***n*** = 188) in the pilot study**. The lines in the bars represent medians and bars indicate the range between 75th and 25th percentile. Error bars show the range of masculinity-femininity scores for non-outliers. Dots represent outlying values (1.5 *SD* above/below median).

We found the expected bimodal distribution of the TMF scores. Men and women differed significantly in terms of the scale mean, *M*_*male*_ = 2.56 (*SD* = 0.80), *M*_*female*_ = 5.28 (*SD* = 0.76), *t*_(304)_ = −29.83, *p* < 0.001, and on every item, all *t*s_(287)_ > −10.41, all *p*s < 0.001. With the exception of two outlier individuals, the overlap between men's and women's scores was very small (see Figure 1). According to Kolmogorov-Smirnov statistics, the TMF scores were normally distributed for men (*Z* = 0.99, *p* = 0.28) and women (*Z* = 0.78, *p* = 0.58). Predicting gender by the TMF scores in a logistic regression analysis was 97% accurate [*B* = 4.43, *SE* = 0.69, $\chi_{\left(1\right)}^{2}=41.38$, *p* < 0.001; Nagelkerke's *R*^2^ = 0.92; Model $\chi_{\left(1\right)}^{2}=$ 347.87, *p* < 0.001].

Taken together, confirming Hypothesis 1, we found that the TMF tapped a one-dimensional construct which is in line with lay ascriptions and previous findings regarding the items masculine and feminine. All factor loadings were similar (Δ < 0.1), so that an unweighted additive overall score was justified (Bortz and Döring, 2006). Its single items represented the overall scale very well and were strongly connected to each other. Hence, no item had to be excluded due to low item-specific homogeneity (Bortz and Döring, 2006). Moreover, confirming Hypothesis 2, the TMF was shown to discriminate between women and men at the scale and at the item level. Therefore, we kept all items in the TMF.

## Study 1

The aim of Study 1 was to test the one-dimensionality, reliability, and validity of the TMF. We used a known-groups approach, with lesbians, bisexual, and straight women, to assess which of several gender-related scales is best in differentiating between these groups. In addition to the TMF, we used the BSRI as the gold standard in gender-related assessment. However, we also used the Gender Role Behavior Scale (GRB, Athenstaedt, 2003) and a newly created measure of childhood gender conformity (see Appendix in Supplementary Material). Moreover, an Implicit Association Test (IAT, Greenwald et al., 1998) was used to measure implicit associations of self with masculine vs. feminine.

We assumed that the TMF would reflect a one-dimensional masculinity-femininity construct (Hypothesis 1). Furthermore, we expected that on each measure, straight women would score higher on femininity and/or lower on masculinity as compared to lesbians (Hypothesis 3a). Bisexual women should score in between (Hypothesis 3b). Additionally, on an IAT (see below for details), we assumed straight women to associate more with feminine and less with masculine than lesbians (Hypothesis 6). Gender-related measures should be correlated with each other (Hypothesis 7), and scores on each measure should predict sexual orientation. We also tested the incremental validity of the TMF over the other measures. The TMF should predict sexual orientation better than other gender-related scales (Hypothesis 8). Finally, the TMF should measure a higher-order factor “core” masculinity-femininity that mediates effects of sexual orientation on other gender-related scales (Hypothesis 5). If women differ in masculinity-femininity based on their sexual orientation, indirect effects of the more specific masculinity-femininity related measures via the TMF on sexual orientation should be observed.

### Method

#### Participants

Participants were 126 women from Germany and Luxembourg who took part in the study, voluntarily without compensation. Their age ranged from 19 to 47 years (*M* = 31.13, *SD* = 8.52). Participants were recruited either at the University of Trier or by a snowball technique. Given their scores on a Kinsey-like scale, they were divided into three groups of 47 straight women (Kinsey scores: 6–7), 32 bisexual women (3–5), and 47 lesbians (1–2). Most of the women were well educated, with 50% possessing university entrance qualifications and 40% holding a university degree. With α = 0.05 and *N* = 126, based on Cohen's (1977) conventions, medium-size regression coefficients (*f* ^2^ = 0.35) could be detected with a statistical power of 1 − β = 0.95 in a multiple linear regression with six predictors (Faul et al., 2007).

#### Materials

##### Implicit association test

In essence, IATs comprise two combined tasks in which stimuli that belong to four concepts are mapped onto two responses in different ways. IATs are based on the following idea: If someone is able to react relatively fast when two concepts share a response, these concepts appear to be associated for that person. In detail, stimuli were presented that represent the concepts *self, others, feminine*, and *masculine*. In one task, stimuli representing *self* or *feminine* required one response, and stimuli representing *others* or *masculine* required the other response (e.g., left vs. right key press). In the other task, stimuli representing *self* or *masculine* required one response, and stimuli representing *others* or *feminine* required the other response. A person considering herself feminine should be able to react faster in the self-feminine/others-masculine than in the self-masculine/others-feminine task.

We labeled one dimension for the IAT “typically feminine” vs. “typically masculine.” The associated attributes presented were *feminine, female* vs. *masculine, male* (in German: *feminin, weiblich; maskulin, männlich*, see Steffens et al., 2008). The other dimension was “self” vs. “others.” The stimuli on that dimension were synonyms of the superordinate concepts (*me, self* vs. *you, others*; in German: *Ich, Selbst; Du, Andere*). Participants were informed that concepts would be displayed throughout at the top left or right screen corner. Their task during the IAT would be to sort words belonging to these concepts by pressing the respective response key on the left or right as quickly as possible. A stimulus word would appear (e.g., feminine) after which participants would respond by pressing the appropriate key (e.g., left for *typically feminine*). The word would then be replaced by the next stimulus (e.g., *me*). Participants would again select the appropriate key (e.g., left for *self*). Each crucial, combined task consisted of four blocks of 62 trials. The order of the eight stimuli was randomized within each block, and the same eight stimuli were presented over and over. The reaction-stimulus interval was 200 ms. Missing reactions and errors led to an appropriate visual feedback (e.g., in case of errors, F! was shown for 200 ms). Participants received feedback on errors and reaction times after each block (e.g., given 10% errors or more: “You committed many errors. Please react more slowly and more correctly.”).

The IAT effect was computed similar to the IAT D effect (Nosek et al., 2005, except that no “error penalty” was used, see Steffens et al., 2008): Specifically, the reaction time difference between the self-feminine/others-masculine and the self-masculine/others-feminine task was computed and divided by each individual's standard deviation across both tasks. In order to avoid artificially high scores obtained with very long scales, internal consistency was estimated based on the average reaction time difference in reaction to each of the eight stimuli. In other words, the IAT was treated as an eight item scale (following Steffens and Buchner, 2003). All internal consistencies are presented in Table 2.

**Table 2:** **Internal Consistencies (Cronbach's α, with number of items) and Correlations between Measures in Study 1**.

	**Alpha (items)**	**TMF −M**	**BSRI −F**	**BSRI −M**	**GRB −F**	**GRB −M**	**CGRB −F**	**CGRB −M**	**IAT effect**
TMF-F	0.90 (6)	−0.85	0.42	(−0.07)	0.41	−0.51	0.71	−0.65	0.30
TMF-M	0.89 (6)		−0.30	(0.17)	−0.37	0.44	−0.60	0.57	−0.28
BSRI-F	0.83 (10)			(−0.08)	0.40	(−0.06)	0.21	(−0.11)	0.18
BSRI-M	0.78 (10)				(0.05)	(0.12)	(0.01)	(0.06)	−0.24
GRB-F	0.87 (29)					(−0.11)	0.40	−0.25	0.18
GRB-M	0.83 (23)						−0.48	0.47	(−0.10)
CGRB-F	0.88 (5)							−0.90	0.31
CGRB-M	0.88 (5)								−0.31
IAT effect	0.93 (8)								

*All correlations are statistically significant at α ≤ 0.05 except for those in parentheses. Abbreviations: TMF, Traditional Masculinity-Femininity; BSRI, Bem Sex Role Inventory; GRB, Gender-Role Behavior; IAT effect: Differences in the implicit association test (IAT) between the self-masculine/others-feminine and the self-feminine/others-masculine task. Endings indicate masculinity (−M) und femininity (−F) scales. Higher scores indicate higher masculinity on masculinity scales and higher femininity on femininity scales*.

##### Bem sex-role inventory

We translated the English short version of the BSRI (Bem, 1979) into German. It consisted of 30 items, 10 for the Masculinity Scale (e.g., self-reliant, ambitious), 10 for the Femininity Scale (e.g., warm, tender), and 10 neutral items with a 7-point scale anchored 1 (*never applies*) to 7 (*always applies*). Participants were asked to rate the extent to which the given traits were adequate to describe them.

##### Traditional masculinity-femininity

The TMF was used as described in the Section Scale Construction with two unipolar dimensions, masculinity and femininity (12 items overall, see Appendix in Supplementary Material).

##### Childhood gender role behavior (CGRB)

Five items were used with a 7-point-scale in order to measure whether participants remembered to have been rather feminine during childhood, or rather typical girls, or not (see Appendix A2 in Supplementary Material). For example, we asked whether they had played with girls and girls' games, and whether they had liked wearing skirts and dresses.

##### Sexual orientation

As indicated in Section Participants, participants' sexual orientation was assessed using participants' responses on the item: “Regarding sexual orientation, I identify as …” (on a Kinsey-like scale, from 1 (*exclusively lesbian*) to 7 (*exclusively straight*). This was also the first item of a translated version of the Assessment of Sexual Orientation Scale (Coleman, 1987). Several additional items were originally used (sexual behavior: gender of partner and ideal partner; sexual fantasies, and emotional bindings). To be consistent with Study 2, we used only the first item to group participants as lesbians (scores 1–2), bisexual women (scores 3–5), and heterosexual women (scores: 6–7). The first item also correlated highly with the overall scale (*r* = 0.95), corroborating the decision to use only one item.

##### Gender role behavior scale

Participants rated themselves on a 7-point scale ranging from 1 (*not at all typical*) to 7 (*very typical*) on 52 everyday typically feminine or masculine behaviors (GRB, Athenstaedt, 2003; e.g., “watch soap operas,” “change light bulbs”).

#### Procedure

Participating students were tested at the University of Trier in a lab cubicle equipped with an iMac. The participants recruited via the snowball technique were tested individually in their homes or offices (as they wished) using an iBook. The instructions, the implicit tests, and the questionnaires were presented by a self-composed HyperCard computer program. Initially, participants were asked to report their age, educational background, and size of hometown. Then, they started with the IAT. IAT task order was held constant because of the correlational nature of the study (see e.g., Banse et al., 2001, for discussion). All participants did the self-masculine/others-feminine task first. After the IAT, the questionnaires were presented in the order described in the Materials Section—accordingly, data for the TMF was collected before all other scales. Finally, participants were debriefed and thanked.

### Results

In all analyses in the present article, significance tests were conducted with α = 0.05 and all statistical analyses were done with SPSS 22. One might suggest that all other scales in addition to the TMF used in the present research should also be submitted to factor analyses. However, commonalities of several of them were too low for conducting confirmatory factor analyses. To illustrate, in Study 2 we observed GRB-M (<0.01) and GRB-F (<0.10). Therefore, means of all established gender-related scales were computed according to the scales' theoretical basis as suggested by their authors.

#### Factor analysis

In order to check for one-dimensionality of the TMF, an exploratory PAF with oblique rotation (oblimin: 0) was conducted for all 12 items. Sample adequacy was confirmed by a KMO criterion of 0.86. All items were suitable for factor analysis as indicated by item-specific KMO values >0.77 and moderate to high commonalities (0.50–0.80). Several indicators are in line with the same one-factor solution as in the Pilot Study and in Study 2 below. According to a graphical scree-plot analysis, a one-factor solution was confirmed. There was a steep decline of explained variance from factor one (61%) to factor two (12%). Moreover, the factor matrix showed a strong first factor suggesting all items to measure something similar.

An alternative confirmatory factor analysis with one factor replicating the findings of the Pilot Study yielded an overall explained variance of 57.80% and showed all items to load highly on that factor (positive loadings for femininity items: ≥ 0.70; negative loadings for masculinity items: ≤ −0.67). Taken together, a one-factor solution was indicated. Factor, pattern, and structure matrix for the exploratory factor analysis and factor loadings for the confirmatory factor analysis can be found in Table B1 in Appendix B in Supplementary Material.

#### Group differences

Table 3 shows overall scale means, average scores for each sexual-orientation group, and statistical tests. As expected, lesbians scored lower on TMF femininity and higher on TMF masculinity than bisexual or straight women. All differences between groups were statistically significant (based on a Scheffé test), except that bisexual women did not score significantly higher than straight women on masculinity. On the BSRI, no significant differences between groups were obtained. In contrast, regarding gender-role behavior and childhood behavior, expected differences between lesbians and straight women were obtained. Similarly, the implicit association of self with feminine was stronger in straight women than lesbians, confirming expectations.

**Table 3:** **Overall Scale Means (with ***SD***) and Means per Group, with Statistical Test of Difference (all *df* = 2, 123; with effect size; Tukey HSD) and Correlation with the Sexual Orientation Scale in Study 1**.

	***M***	**Lesbians (*n* = 47)**	**Bisexual women (*n* = 32)**	**Straight women (*n* = 47)**	**Group differences** ($R_{p}^{2}$)
TMF-F	4.24 (1.35)	3.20 (1.05)	4.47 (1.00)	5.13 (1.12)	*F* = 40.19, *p* < 0.001 (0.40) L < B < S
TMF-M	3.49 (1.22)	4.36 (0.90)	3.24 (1.02)	2.78 (1.09)	*F* = 30.31, *p* < 0.001 (0.33) L > B = S
BSRI-F	5.24 (0.75)	5.09 (0.71)	5.38 (0.85)	5.30 (0.69)	*F* = 1.63, *p* = 0.19, *ns*
BSRI-M	4.67 (0.73)	4.69 (0.75)	4.72 (0.83)	4.61 (0.65)	*F* < 1, *ns*
GRB-F	4.38 (0.85)	4.16 (0.80)	4.45 (0.83)	4.57 (0.89)	*F* = 2.94, *p* = 0.06 (0.05) L < S
GRB-M	4.22 (0.86)	4.73 (0.79)	4.09 (0.83)	3.78 (0.68)	*F* = 19.29, *p* < 0.001 (0.24) L > B = S
CGRB-F	3.97 (1.63)	2.93 (1.55)	4.28 (1.37)	4.79 (1.30)	*F* = 21.40, *p* < 0.001 (0.26) L < B = S
CGRB-M	4.29 (1.52)	5.24 (1.54)	3.98 (1.25)	3.56 (1.16)	*F* = 19.93, *p* < 0.001 (0.25) L > B = S
IAT effect	0.67 (0.31)	0.59 (0.31)	0.65 (0.31)	0.76 (0.30)	*F* = 3.81, *p* < 0.03 (0.06) L < S

*All scales theoretically range from 1 to 7, except for the IAT effect that is similar to an effect size of the stronger self-feminine as opposed to self-masculine association. Abbreviations of tests: TMF, Traditional Masculinity-Femininity; BSRI, Bem Sex Role Inventory; GRB, Gender-Role Behavior; IAT effect: Differences in the implicit association test (IAT) between the self-masculine/others-feminine and the self-feminine/others-masculine task. Endings indicate masculinity (−M) und femininity (−F) scales. Higher scores indicate higher masculinity on masculinity scales and higher femininity on femininity scales. Abbreviations of groups: L, Lesbians; B, bisexual women; and S, straight women*.

#### Bivariate correlations

Table 2 shows bivariate correlations, along with internal consistencies. Internal consistencies of all measures were excellent, with the lowest score obtained for BSRI masculinity. A noteworthy correlation was a strong negative one between the TMF factors masculinity and femininity, suggesting that a one-dimensional measure could be sufficient. In line with the large negative correlation, people who judged themselves as “moderately feminine” (i.e., ticked the value 4) tended to also judge themselves as “moderately masculine” (i.e., ticked 4 again). Hence, we recoded all masculine items and then averaged all items of the TMF to obtain a supplementary measure, TMF total. TMF masculinity and femininity correlated in the expected direction with all other measures except for BSRI masculinity. BSRI masculinity did not correlate significantly with any other measure, suggesting that it measured something different from all other measures of masculinity in the study. All other correlations were in the expected direction. Of particular interest, the implicit association of self-feminine correlated positively with TMF femininity and negatively with TMF masculinity, as expected. Similar, but somewhat weaker relations were obtained between the IAT and most other measures.

#### Predicting sexual orientation

In order to test whether lesbians, bisexual, and straight women would be classified correctly based on the different measures of masculinity-femininity, we carried out an ordinal regression analysis. As predictor variables, the masculinity and femininity scores of BSRI, GRB, and CGRB were entered. In addition, TMF total and the IAT effect were used as predictors. The overall model was statistically significant, χ_(8)_ = 72.01, *p* < 0.001, Nagelkerke's *R*^2^ = 0.49. The significant predictors were TMF total scores [*B* = 1.17, *SE* = 0.27, χ^2^_(1)_ = 19.30, *p* < 0.001] and masculine everyday behavior [*B* = −0.69, *SE* = 0.27, χ^2^_(1)_ = 6.65, *p* = 0.01]. None of the other predictors was significant, *p*s > 0.21. Thus, based on their self-assessment on the TMF as masculine-feminine and based on the masculine everyday behaviors participants said they carried out, they could be classified quite well as lesbians, bisexual, or straight women.

#### Mediation analyses

**Figure 2:**
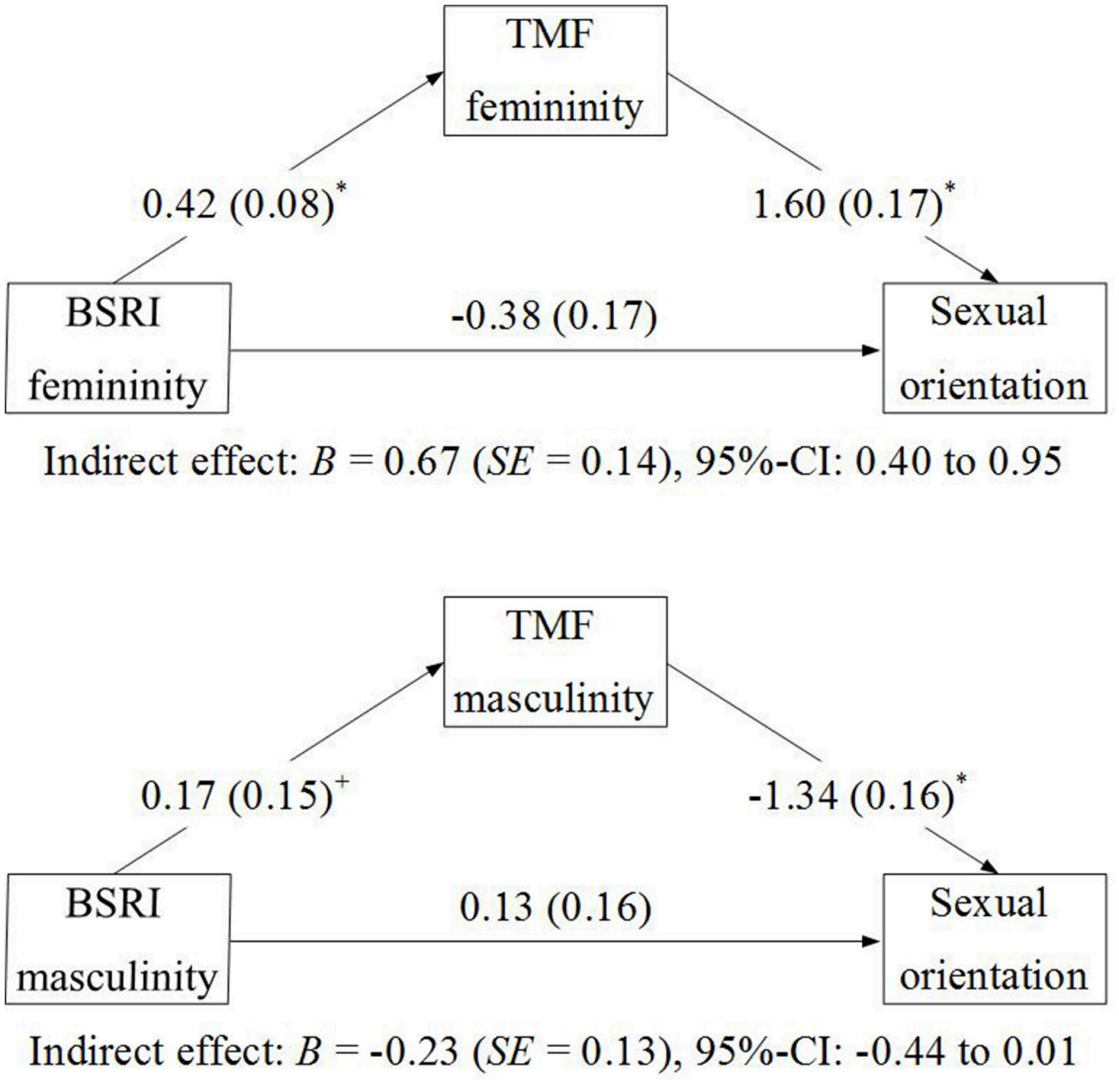
**Mediation of the relation between BSRI and sexual orientation by the TMF**.

**Figure 3:**
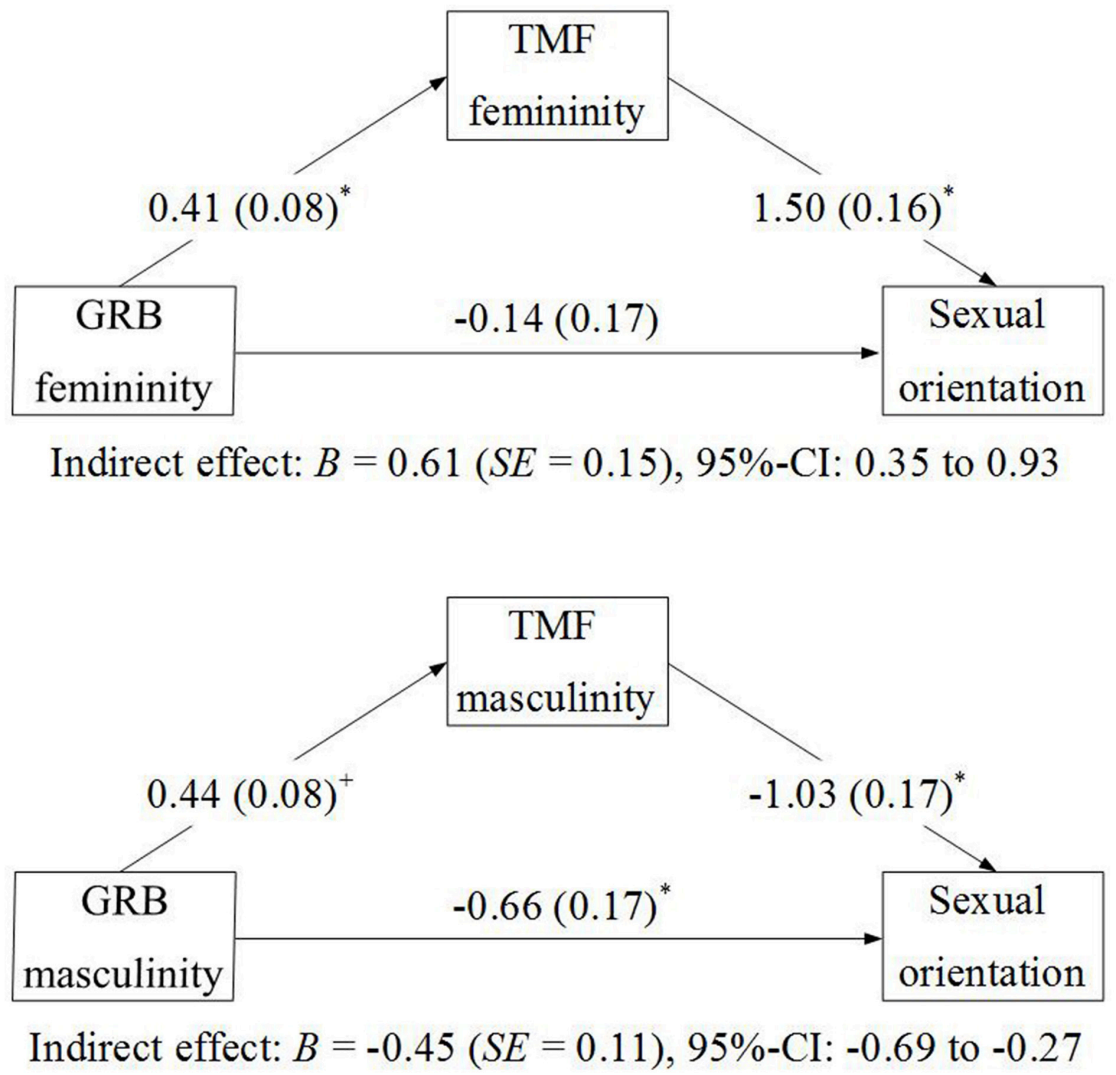
**Mediation of the relation between GRB and sexual orientation by the TMF**.

Based on the regression approach suggested by Hayes (2013), we tested whether there are indirect effects of the BSRI and GRB dimensions on sexual orientation via the respective TMF dimensions. Because this approach needs a continuous dependent variable, in contrast to all other analyses in the present paper, we did not use the classification as lesbian, bisexual, or straight in this case, but the continuous Kinsey-like scale with scores ranging from 1 to 7. Figures 2, 3 summarize the findings. Statistically significant effects of BSRI femininity and GRB femininity on TMF femininity were observed, and also of GRB masculinity and of BSRI masculinity (by trend) on TMF masculinity. TMF masculinity and femininity were related with sexual orientation in expected ways (in line with the findings reported in Table 3). Bootstrapping analyses, using 10,000 Bootstrap re-samples, demonstrated that the indirect effects of BSRI femininity, GRB femininity, and BSRI femininity on sexual orientation via the TMF were statistically significant (i.e., none of the bias-corrected 95% confidence intervals included 0). The indirect effect of BSRI masculinity via TMF masculinity missed the preset criterion of statistical significance. Only one direct effect was significant in addition to the indirect effect: Whereas all other findings were in line with the interpretation of full mediation via the TMF, masculine everyday behavior was still related to sexual orientation when the TMF was included in the equation. This suggests that the TMF mediated the relationship between sexual orientation and masculine behavior only partially.

### Summary of findings

In Study 1, we found that the reliabilities of both the femininity and the masculinity subscales of the TMF were high. Moreover, they correlated so strongly (in a negative way) that one may also conceive of the scale as one-dimensional, ranging from masculinity to femininity. We found several pieces of evidence for the validity of the scale. First, it correlated in the expected directions with all other measures of masculinity and femininity that we used, except for BSRI masculinity, which largely confirms Hypothesis 7. Feminine traits as well as masculine and feminine behaviors can be predicted quite well from scores on the TMF. The strongest correlations were obtained with self-rated childhood gender conformity. Notably, confirming Hypothesis 6, correlations with an implicit measure of one's self-feminine vs. self-masculine association were in the expected order of magnitude (e.g., Hofmann et al., 2005) and higher than those of the implicit measure with any of the trait or behavior self-ratings. Additionally, the TMF was related to participants' sexual orientation more strongly than any other measure (see ANOVA results in Table 3), with lesbians reporting lower femininity and higher masculinity than bisexual or straight women (confirming Hypothesis 3a and b). When predicting participants' sexual orientation from the masculinity and femininity measures, neither feminine, nor masculine traits, nor feminine everyday behavior, nor the self-feminine association contributed. Instead, confirming Hypothesis 8, masculine everyday behavior and the TMF were able to predict participants' sexual orientation very well, attesting to the usefulness of two rather new conceptualizations of measuring masculinity and femininity.

Mediation analyses were in line with the idea that feminine traits and feminine everyday behavior differ by sexual orientation because of a globally more feminine gender-role self-concept. This confirms Hypothesis 5. Masculine traits also tend to differ by sexual orientation because of lesbians' globally more masculine gender-role self-concept. Further, masculine everyday behavior also differs by sexual orientation because of lesbians' globally more masculine gender-role self-concept, but a direct effect of masculine behavior on sexual orientation remained. A speculative explanation for the latter finding is that it may depend partly on the gender of one's relationship partner which behaviors one carries out. For example, given that couples typically divide housework in ways mirroring traditional gender roles (e.g., Croft et al., 2014; Steffens and Viladot, 2015), a woman considering herself rather feminine may mow the lawn more often when she is in a relationship with a woman than with a man. In other words, in addition to personal preferences, the presence or absence of other-gender people in the household who choose to take care of certain chores may determine which chores one does (i.e., typically male everyday behaviors if no man is around).

## Study 2

The aim of Study 2 was to replicate and extend Study 1's findings. We used data of a research project on social perception. As in Study 1, we used a known-groups approach, this time contrasting lesbians, gay men, and straight women and men. With the exception of small adjustments, gender-related scales were identical to Study 1. However, this time we used a different adjective-based instrument than the BSRI, namely the GEPAQ, the German version (Runge et al., 1981) of the Extended Personal Attributes Questionnaire (Spence et al., 1978). For determining criterion validity, we also focused on other features. Participants were instructed to provide information regarding frequency of contact with lesbians/gay men and straight people. Moreover, characteristics of participants' voice pitch were collected as well as evaluations from independent judges on whether participants' voices sounded straight or gay/lesbian and whether their faces looked straight or gay/lesbian. In order to determine the TMF's test-retest reliability, we re-invited male participants after 1 year (for female participants no contact data were available).

We expected highest masculinity/lowest femininity scores for straight men, followed by gay men, lesbians, and straight women, implying lowest masculinity/highest femininity for straight women (Hypotheses 2, 3, and 4). We expected gender-related characteristics to correlate moderately with the TMF (Hypothesis 7) and we assumed the TMF to predict sexual orientation better than the other gender-related scales (Hypothesis 8). Furthermore, we assumed that participants with higher gender-conform scores on the TMF would report less contact with lesbians and gay men (Hypothesis 10), would show rather gender stereotypical voice pitch characteristics (Hypothesis 11), and would be more likely to be rated as straight (Hypothesis 9). A moderate 1-year reliability was expected (Hypothesis 12) as well as a moderate predictive validity for the second measurement of gender-related features (Hypothesis 13).

### Method

#### Participants

Overall 111 German participants attended the study at the first measurement point. Their age ranged from 19 to 30 years (*M* = 24.2, *SD* = 2.5). Participants were recruited at the University of Jena, the Technical University of Berlin, and on lesbian/gay dating websites. Based on their Kinsey-like scale scores, 15 participants who rated themselves as bisexual were excluded from further analyses because of the small group size. Among the remaining 96 participants, there were 24 lesbians (Kinsey scores: 1–2), 21 straight women (6–7), 25 gay men (1–2), and 26 straight men (6–7). Most participants were well educated, 60% possessing a university entrance qualification and 35% a university degree. As a *post-hoc* power analysis indicated, given the sample size and α = 0.05, between medium (0.25) and large (0.40) effects of *f* = 0.35 could be detected in the 2 × 2 ANOVAs below with a statistical power of 1 − β = 0.95.

A total of 37 men attended the post-test. According to their Kinsey-like scale scores 18 identified as gay (1–2) and 19 as straight (6–7). Between those attending the post-test and those who did not, merely one difference was significant after adjusting the significance level for multiple tests. The retest-group reported less contact with straight men during the first data collection [*M*_*retest*_ = 5.76, *M*_*no*−*retest*_ = 6.53, *t*_(49.47)_ = 3.33, *p* = 0.002].

#### Materials

The same measures as in Study 1 were used in the following manner. Because the femininity and masculinity subscales of the TMF were highly correlated, as were subscales of the Childhood Gender-Role Behavior Scale, they were combined to form one dimension each [TMF: 1 (*very masculine*), to 7 (*very feminine*); CGRB: 1 (*I strongly disagree*), 5 (*I strongly agree*)]. Thus, the 6-item-version of the TMF was used. High values on CGRB indicated a high degree of gender conformity. Gender Role Behavior was assessed with a 6-point-scale this time and sexual orientation was measured with one item on a 7-point Kinsey-like scale [(“Regarding sexual orientation, I identify as…”); 1 (*exclusively lesbian/gay*), 7 (*exclusively straight*)]. Moreover, we included the following measures.

##### German extended personal attributes questionnaire

We used the German version (Runge et al., 1981) of the Extended Personal Attributes Questionnaire (Spence et al., 1978). It consists of two independent scales measuring gender-related personality traits. The instrumentality scale (GEPAQ-M) contained eight items describing behaviors more socially desirable for men (e.g., independent), the expressiveness scale (GEPAQ-F) comprised eight items more socially desirable for women (e.g., emotional). Participants were instructed to indicate on a 5-point Likert-type scale (1 = non independent/not emotional, 5 = very independent/very emotional) the extent to which they felt each item described them.

##### Contact measures

In order to estimate the composition of participants' social environment, we measured current contact to same-gender lesbian/gay and straight people with one item each. The participants should “indicate how often you have contact to homosexual and heterosexual women/men” on a 7-point scale ranging from 1 (*never*) to 7 (*always*).

##### Voice pitch characteristics

To describe participants' voice pitch (i.e., the auditory correlate of fundamental frequency) distributions in spontaneous speech, we used three measures. Mean fundamental frequency (f0) indicates the average voice pitch, f0 standard deviation is a measure for voice pitch variability, and f0 range is used to evaluate voice pitch range. For computing f0 range, we computed the difference between the f0 97.5th percentile (estimator of the upper voice pitch boundary) and f0 2.5th percentile (estimator of the lower voice pitch boundary).

##### Perceived straightness

Participants' voices, facial photographs, and the combinations of both voices and faces had been rated as either “heterosexual” or “homosexual” by 101 judges (65 women, 31 men; age *M* = 28.0), participating in a different study (for details see Kachel et al., unpublished manuscript). To receive a relative measure of “heterosexual” judgments, all “heterosexual” responses were summed for each participant and divided by the number of judgments. Hence, higher scores indicate higher perceived straightness.

#### Procedure

At first measurement, participants filled out an online questionnaire in which all psychological and sociodemographic characteristics were collected. The order of psychological instruments was TMF, CGRB, contact to girls and boys during childhood, GRB, GEPAQ, Kinsey-like scale, and finally current contact to same-gender lesbians/gay men and straight people. In the second step, they were invited to a speech lab to provide recordings of spontaneous spoken speech and text reading as well as a photograph of their face. The sampling of women took place in a phonetic laboratory in the Center of General Linguistics in Berlin and was done by a female investigator, whereas the sampling of men took place at a phonetic laboratory of the University of Jena and was done by a male investigator. Voice pitch characteristics were measured on the basis of spontaneous speech. In the last step we asked 101 judges to rate speech recordings, facial photographs, and the combination of both dichotomously regarding sexual orientation for a randomly selected subset of 18 lesbians, gay men, straight women, and men, respectively (Kachel et al., unpublished manuscript). For the rating of speech recordings, we used the same read sentence for all target persons (“It has been quite a long day,” German: “*Der Tag ist sehr lang geworden*.”) in order to hold the conditions constant for every target and to control for the phonetic composition of the utterance.

Male participants were re-invited after 1 year to the phonetic laboratory of the University of Jena. Before speech recordings they were asked to fill out an online questionnaire containing several gender-related scales including the 6-items version of the TMF, the GEPAQ-M, and the GEPAQ-F.

### Results

All results refer to the first measurement except for those that are explicitly indicated to belong to second measurement.

#### Factor analysis

In order to test whether the TMF scale is one-dimensional, an explorative factor analysis with PAF was conducted. It replicated all findings of the pilot study. In detail, a KMO criterion of 0.86 indicated that the sample was appropriate. All items were suitable for factor analysis (item-specific KMO values >0.81; commonalities:0.54–0.83). According to a graphical scree-plot analysis, a one-factor solution was confirmed. There was a steep decline of explained variance from factor one (71%) to factor two (13%). Each item was represented very well by this factor (loadings >0.73).

An additional exploratory factor analysis with PAF of participants at second measurement replicated the findings indicating a one-dimensional factor structure. In detail, a KMO criterion of 0.76 indicated that the sample was appropriate. All items were suitable for factor analysis because of item-specific KMO values >0.69 and moderate to high commonalities (0.42–0.69). The one-factor solution was confirmed by graphical scree-plot analysis. There was a steep decline of explained variance from factor one (60%) to factor two (14%). Each item was represented very well by this factor (loadings >0.65).

#### Differences on gender-related scales based on gender and sexual orientation

Which of the gender-related instruments are able to predict a person's gender and sexual orientation? In order to answer this question, for all gender-related instruments separate 2 × 2 ANOVAs with the two between-subject factors gender and sexual orientation were computed. Simple-effects tests with Bonferroni adjustment were added. Table 4 shows main and interaction effects as well as mean scores for all gender-related instruments separately for lesbians, straight women, gay, and straight men.

**Table 4:** **Group-Specific Means (with ***SD***) on Gender-Related Scales and ANOVA Results regarding Sexual Orientation and Gender in Study 2 at First Measurement**.

	**Women**		**Men**		**SexOr (*F*, *p*, η^2^)**	**Gender (*F*, *p*, η^2^)**	**Gender × SexOr (*F*, *p*, η^2^)**
	**L^1^**	**S^2^**	**G^3^**	**S^4^**			
TMF	^a^4.54 (1.15)	^b^5.36 (0.72)	^c^3.49 (0.87)	^d^2.51 (0.98)	0.15, 0.703, 0.00	100.54, < 0.001, 0.52	21.42, < 0.001, 0.19
GEPAQ-M	^a^3.29 (0.45)	^a^3.23 (0.40)	^a^3.31 (0.70)	^a^3.41 (0.55)	0.02, 0.886, 0.00	0.79, 0.376, 0.01	0.47, 0.494, 0.01
GEPAQ-F	^a^4.04 (0.55)	^a^4.18 (0.51)	^a^4.06 (0.48)	^b^3.68 (0.48)	1.48, 0.227, 0.02	5.36, 0.023, 0.06	6.35, 0.013, 0.07
GRB-M	^a^3.59 (0.78)	^a^3.45 (0.79)	^a^3.42 (0.66)	^b^4.17 (0.78)	3.08, 0.082, 0.03	1.94, 0.167, 0.02	6.41, 0.013, 0.07
GRB-F	^a^3.91 (0.76)	^b^4.63 (0.45)	^a^3.57 (0.77)	^c^3.17 (0.78)	1.03, 0.313, 0.01	37.11, 0.001, 0.29	14.42, < 0.001, 0.14
CGRB	^a^3.04 (1.10)	^a^3.38 (0.92)	^a^3.22 (0.67)	^b^4.32 (0.52)	18.05, < 0.001, 0.16	11.00, 0.001, 0.11	4.99, 0.028, 0.05

*TMF: 1-7, Traditional Masculinity-Femininity; GEPAQ: 1-5, German Extended Personality Attributes Questionnaire; GRB: 1-6, Gender-Role Behavior; and CGRB: 1-5, Childhood Gender-Role Behavior. Endings indicate masculinity (−M) und femininity (−F) scales. Higher scores indicate higher masculinity on masculinity scales, higher femininity on femininity scales and TMF, and higher gender-conformity on CGRB. According to a Levene test, the assumption of homogeneity of variance was violated for GRB-F and CGRB. Superscripted letters in mean columns refer to groups based on simple-effect findings. Groups sharing the same letter do not differ significantly from each other at α ≤ 0.05*.

1 *Lesbians n = 25*.

2 *Straight women n = 26*.

3 *Gay men n = 24*.

4 *Straight men n = 21*.

On the TMF, we found an interaction of gender and sexual orientation, *F*_(1, 92)_ = 21.42, *p* < 0.001, $\eta_{p}^{2}$ = 0.19, as well as a main effect of gender *F*_(1, 92)_ = 100.54, *p* < 0.001, $\eta_{p}^{2}$ = 0.52. Both effects explained more variance in the TMF than in all other gender-related instruments in this study. Because straight women and men conform to gender roles more than lesbians/gay men, stronger gender differences should be expected between straight women and men than between lesbians and gay men. Hence, comparing lesbians and gay men constituted a stricter test of all scales. Although the TMF mean differences between straight women and men were more distinct (Δ*M* = 2.85), lesbians and gay men significantly differed, too (Δ*M* = 1.05). In short, the TMF showed the expected mean differences between all groups, it was the only scale in this study that was able to detect differences between lesbians and gay men, and it showed the largest mean difference between straight women and men.

**Figure 4:**
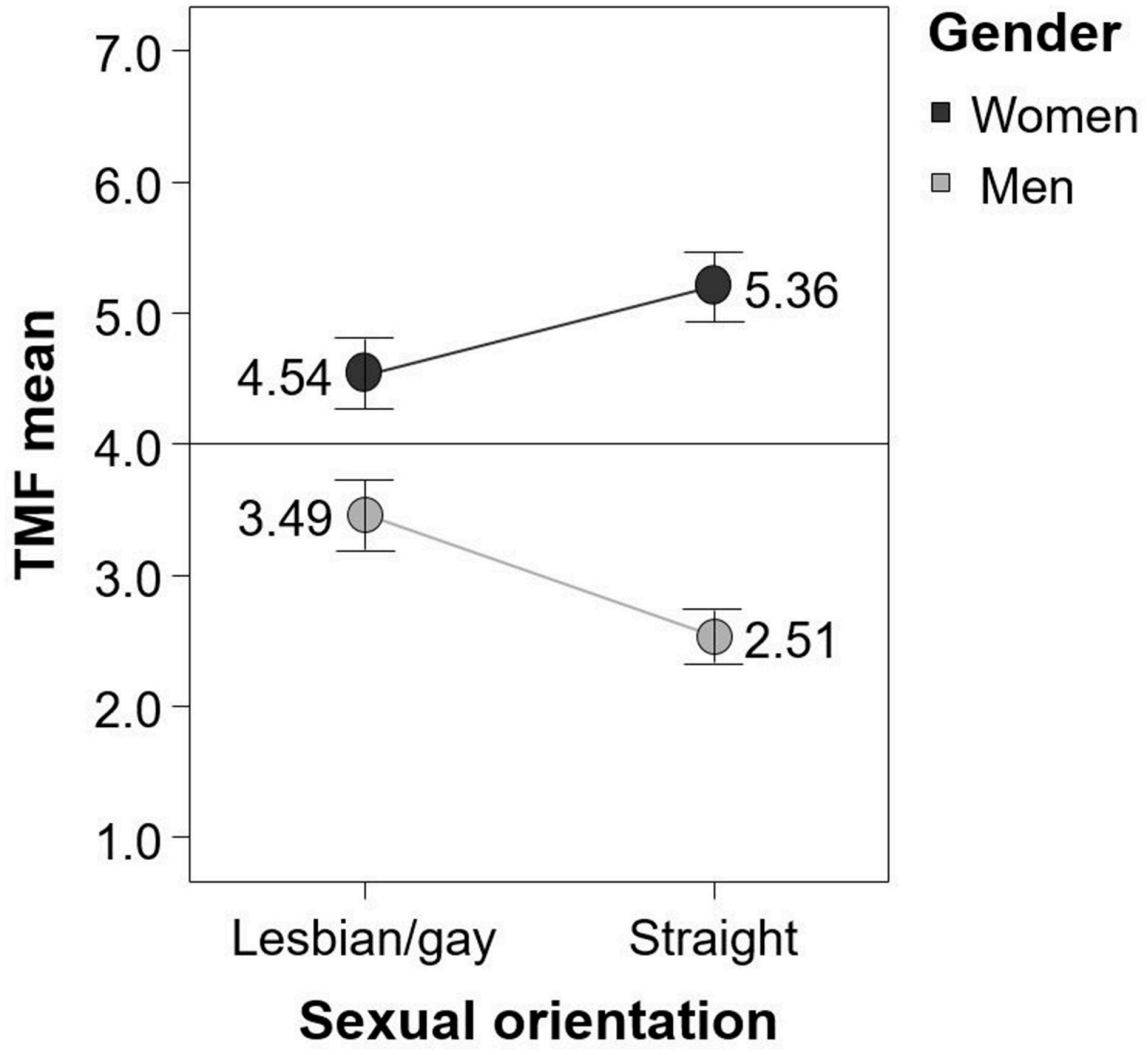
**Mean TMF scores separately for gender and sexual orientation**. Error bars represent standard errors of means.

Furthermore, the TMF differentiated the groups as expected (see Figure 4). Lesbians and straight women were on average clearly located on the scale's side that is associated with femininity (scores > 4) and gay and straight men's mean values were connected to masculinity (scores < 4). Additionally, the TMF was best in predicting gender on the basis of scale scores as can be seen in Table 5 in which results of binary logistic regression models for all gender-related scales are shown. Correct gender classification rate for the TMF was 80%. Almost identical percentages of women and men were correctly classified. Compared to all other measures under investigation, the TMF seemed to be the most precise instrument to differentiate between women and men regardless of their sexual orientation.

**Table 5:** **Results of Binary Logistic Regression Models in Predicting Participants' Gender based on Different Gender-Related Instruments in Study 2 at First Measurement**.

	**Percentage of correct classifications**			***B***	***SE***	**$\chi_{\left(1\right)}^{2}$**	***p***
	**Overall**	**Men**	**Women**				
TMF	80.2	80.4	80.0	1.67	0.33	25.50	<0.001
GEPAQ-M	53.1	82.4	20.0	−0.35	0.39	0.79	0.374
GEPAQ-F	60.4	68.6	51.1	0.90	0.42	4.64	0.031
GRB-M	49.0	64.7	31.1	−0.36	0.27	1.81	0.179
GRB-F	70.8	68.6	73.3	1.42	0.32	19.71	<0.001
CGRB	60.4	72.5	46.7	−0.69	0.24	8.23	0.004

*TMF, Traditional Masculinity-Femininity; GEPAQ, German Extended Personality Attributes Questionnaire; GRB, Gender-Role Behavior; and CGRB, Childhood Gender-Role Behavior. Endings indicate masculinity (−M) und femininity (−F) scales*.

#### Replication of findings from the female sample

##### Group differences in women's sample

Regarding TMF, group differences in women's sample were already mentioned above. As in Study 1, straight women described themselves as more feminine compared to lesbians on the GRB-F. However, in contrast to Study 1, other gender-related scales (GRB-M and CGRB) were not able to differentiate women regarding their sexual orientation (see Table 4). Means were particularly close together for adjective-based gender-related instruments such as the GEPAQ.

##### Bivariate correlations

Reliabilities and correlations on all gender-related instruments can be seen in Table 6. Three out of five correlations with the TMF were significant. Besides the GRB-F there was also a correlation with gender-conforming childhood-experiences (CGRB) and with the exchanged adjective-based masculinity-scale (GEPAQ-M). The correlations for the first two instruments were in the expected direction: The more feminine the women rated themselves on the TMF, the higher their scores on behavior-based femininity (GRB-F) and childhood gender-conformity (CGRB). However, the TMF correlated positively with the GEPAQ-M, which is counterintuitive. We believe that this attests to deficiencies in the GEPAQ-M, along with its low reliability. Moreover, after adjusting the significance level according to the Bonferroni formula, the correlation was not significant anymore.

**Table 6:** **Reliabilities and bivariate correlations of gender-related scales for women and men in Study 2 at first measurement**.

	**TMF**	**GEPAQ-F**	**GEPAQ-M**	**GRB-F**	**GRB-M**	**CGRB**
TMF	0.86/0.89	(0.28)	0.34	0.47	(−0.27)	0.54
GEPAQ-F	0.59	0.77/0.64	(0.04)	(0.29)	(0.21)	(0.27)
GEPAQ-M	−0.38	(−0.21)	0.51/0.73	(−0.03)	(−0.09)	(0.12)
GRB-F	0.31	0.46	(0.19)	0.88/0.91	(0.29)	0.47
GRB-M	−0.45	−0.26	0.42	(0.20)	0.83/0.88	(−0.14)
CGRB	−0.55	−0.41	(0.18)	−0.35	0.37	0.82/0.73

*Correlations for women sample are presented above, for men sample below the diagonal. Internal consistencies are depicted in the diagonal with the values before the slash referring to women and after the slash referring to men. All correlations are statistically significant at α ≤ 0.05 except for those in parentheses. Abbreviations: TMF, Traditional Masculinity-Femininity; GEPAQ, German Extended Personality Attributes Questionnaire; GRB, Gender-Role Behavior; and CGRB, Childhood Gender-Role Behavior. Endings indicate masculinity (−M) und femininity (−F) scales. Higher scores indicate higher masculinity on masculinity scales, higher femininity on femininity scales and TMF, and higher gender-conformity on CGRB*.

##### Predicting sexual orientation

Can the TMF predict women's sexual orientation better than other measures? We added the TMF in the last step of a binary regression model. Results can be seen in Table 7. In contrast to Study 1, the TMF did not outperform all other measures. Only the GRB-F was found to predict women's sexual orientation. However, when GRB-F was not included in the regression model, the TMF was the only significant predictor of sexual orientation in the model, *B* = 1.25, *SE* = 0.50, χ^2^_(1)_ = 6.19, *p* = 0.013.

**Table 7:** **Stepwise, logistic regression analysis for predicting women's sexual orientation based on gender-related scales in study 2 at first measurement**.

	**Step 1**	**Step 2**	**Step 3**
**Predictors:**	***B (SE)* Exp(B)**	***B (SE)* Exp(B)**	***B (SE)* Exp(B)**
GEPAQ-F	[0.50 (0.58) 1.66]	[0.39 (0.76) 1.48]	[0.72 (0.92) 2.05]
GEPAQ-M	[−0.37 (0.72) 0.69]	[−0.86 (0.87) 0.43]	[−0.95 (0.94) 0.39]
GRB-F		3.01 (0.97) 20.30	3.69 (1.38) 40.15
GRB-M		−1.13 (0.56) −0.52	[−1.43 (0.80) 0.24]
TMF			[0.35 (0.66) 1.42]
CGRB			[−0.90 (0.58) 0.41]
	$\chi_{\left(2\right)}^{2}$ = 1.01, *p* = 0.605, *R*^2^ = 0.03, 56%	$\chi_{\left(2\right)}^{2}$ = 18.68, *p* < 0.001, *R*^2^ = 0.47, 73%	$\chi_{\left(2\right)}^{2}$ = 2.99, *p* = 0.224, *R*^2^ = 0.53, 78%

*Chosen method was “Forward: Wald” in each block. R^2^ means Nagelkerke's R^2^. Percentage values refer to correctly classified lesbian and straight women. All correlations are statistically significant at α = 0.05 except for those in brackets. TMF, Traditional Masculinity-Femininity; GEPAQ, German Extended Personality Attributes Questionnaire; GRB, Gender-Role Behavior; and CGRB, Childhood Gender-Role Behavior. Endings indicate masculinity (−M) und femininity (−F) scales. Higher scores indicate higher masculinity on masculinity scales, higher femininity on femininity scales and TMF, and higher gender-conformity on CGRB*.

#### Comparisons within men

The same analyses were computed for the male subsample.

##### Group differences

As indicated in Table 4, all differences in the male subsample were in the expected directions. Straight men showed higher masculinity/lower femininity on each gender-related instrument than gay men except for the GEPAQ-M, where no significant difference was detected. The TMF (Δ*M* = 1.05) and the CGRB (Δ*M* = 1.10) were similarly able to predict sexual orientation.

At second measurement, gay and straight men differed more strongly on the TMF [*M*_*gay*_ = 3.85, *M*_*straight*_ = 2.60, *t*_(35)_ = 4.70, *p* < 0.001]. However, in contrast to the first measurement the GEPAQ-F was not able to discriminate between both groups, *M*_*gay*_ = 4.02, *M*_*straight*_ = 3.68, *t*_(35)_ = 1.83, *p* = 0.075. The GEPAQ-M remained non-significant, *M*_*gay*_ = 3.46, *M*_*straight*_ = 3.56, *t*_(35)_ = −0.51, *p* = 0.61.

##### Bivariate correlations

All correlations with the TMF were significant (all |*r*| > 0.31, all *p* < 0.028) and in the expected directions (see Table 6).

##### Predicting sexual orientation

As for the female subsample, the TMF did not predict sexual orientation better than other measures when it was added in the last step of a binary regression model (see Table 8). CGRB and GRB-M were the measures most closely related to sexual orientation. This could be interpreted as suggesting that TMF does not contribute at all to explaining sexual orientation. Moreover, one could be interested in the direct comparison of TMF and GEPAQ in explaining sexual orientation. To answer these questions, in a supplementary binary regression model, only adjective-based scales were included as predictors. In that analysis, TMF was the only significant predictor of sexual orientation, *B* = −0.89, *SE* = 0.41, $\chi_{\left(1\right)}^{2}=4.61$, *p* = 0.032. Taken together, CGRB and GRB-M predicted sexual orientation best, and TMF predicted sexual orientation better than GEPAQ.

**Table 8:** **Stepwise, logistic regression analysis for predicting men's sexual orientation based on gender-related scales in study 2 at first measurement**.

	**Step 1**	**Step 2**	**Step 3**
**Predictors:**	***B (SE)* Exp(B)**	***B (SE)* Exp(B)**	***B (SE)* Exp(B)**
GEPAQ-F	−1.82 (0.72) 0.16	[−0.71 (0.88) 0.49]	[0.64 (1.38) 1.90]
GRB-F		−1.25 (0.61) 0.29	[−1.09 (0.89) 0.34]
GRB-M		1.64 (0.55) 5.17	1.90 (0.86) 6.69
TMF			[−0.25 (0.66) 0.78]
CGRB			3.77 (1.28) 43.39
	χ^2^_(1)_ = 8.17, *p* = 0.004, *R*^2^ = 0.20, 64%	χ^2^_(2)_ = 12.57, *p* = 0.002, *R*^2^ = 0.47, 71%	χ^2^_(2)_ = 19.50, *p* < 0.001, *R*^2^ = 0.73, 86%

*Chosen method was “Forward: Wald” in each block. R^2^ means Nagelkerke's R^2^. Percentage values refer to correctly classified gay and straight men. All correlations are statistically significant at α ≤ 0.05 except for those in brackets. TMF, Traditional Masculinity-Femininity; GEPAQ, German Extended Personality Attributes Questionnaire; GRB, Gender-Role Behavior; and CGRB, Childhood Gender-Role Behavior. Endings indicate masculinity (−M) und femininity (−F) scales. Higher scores indicate higher masculinity on masculinity scales, higher femininity on femininity scales and TMF, and higher gender-conformity on CGRB*.

#### Relations with criterion characteristics

We collected data on several psychological and acoustic criterion characteristics. We computed bivariate correlation coefficients for the TMF with these characteristics in order to test the criterion validity of TMF separately for women (see Table 9) and men (see Table 10). Additionally, correlations for all other gender-related scales included in Study 2 were computed as a comparison.

**Table 9:** **Bivariate correlations of gender-related instruments and criterion characteristics for women in study 2**.

	**TMF**	**GEPAQ**		**GRB**		**CGRB**
		**F**	**M**	**F**	**M**	
Contact to lesbians/gay men	(−0.27)	(−0.03)	(0.20)	(−0.12)	0.30	(−0.10)
Contact to straight wo/men	(0.20)	0.32	(−0.08)	(0.24)	(−0.01)	(0.22)
Voice pitch average	0.41	(0.24)	(0.07)	0.37	(−0.14)	0.46
Voice pitch variability	0.32	(0.14)	(−0.10)	(0.26)	(−0.08)	0.35
Voice pitch range	0.34	(0.19)	(−0.04)	(0.29)	(−0.06)	0.39
Perceived straightness in voice	(0.31)	(0.05)	(−0.01)	(0.20)	(−0.16)	(0.30)
Perceived straightness in face	0.57	(0.09)	(0.03)	(0.29)	(−0.20)	(0.23)
Perceived straightness in voice + face	0.55	(0.11)	(0.01)	(0.31)	(−0.17)	(0.24)

*All correlations are statistically significant at α ≤ 0.05 except for those in parentheses. Abbreviations for column headings: TMF, Traditional Masculinity-Femininity; GEPAQ, German Extended Personality Attributes Questionnaire; GRB, Gender-Role Behavior; and CGRB, Childhood Gender-Role Behavior. Endings indicate masculinity (−M) und femininity (−F) scales. For gender-related instruments higher scores indicate higher masculinity on masculinity scales, higher femininity on femininity scales and TMF, and higher gender-conformity on CGRB. For criterion characteristics higher scores indicate more frequent contact to lesbians/gay men and straight wo/men, higher voice pitch characteristics, and higher perceived straightness*.

**Table 10:** **Bivariate correlations of gender-related instruments and criterion characteristics for men in study 2**.

	**TMF**	**GEPAQ**		**GRB**		**CGRB**
		**F**	**M**	**F**	**M**	
Contact to lesbians/gay men	0.35	0.29	(−0.03)	(0.12)	(−0.08)	(−0.16)
Contact to straight wo/men	(−0.01)	(−0.20)	(00.07)	−0.42	(00.05)	(00.08)
Voice pitch average	(−0.06)	(−0.09)	(0.20)	(0.10)	(−0.01)	(0.00)
Voice pitch variability	(−0.27)	(−0.25)	(0.19)	(−0.17)	(0.23)	0.28
Voice pitch range	(−0.08)	(−0.22)	(0.12)	(−0.07)	(−0.10)	(0.05)
Perceived straightness in voice	−0.34	(−0.19)	(0.04)	(−0.04)	(0.14)	(0.22)
Perceived straightness in face	−0.38	−0.45	(0.17)	−0.47	(0.14)	0.39
Perceived straightness in voice + face	−0.47	−0.49	(0.17)	−0.42	(0.21)	0.48

*All correlations are statistically significant at α ≤ 0.05 except for those in parentheses. Abbreviations for column headings: TMF, Traditional Masculinity-Femininity; GEPAQ, German Extended Personality Attributes Questionnaire; GRB, Gender-Role Behavior; and CGRB, Childhood Gender-Role Behavior. Endings indicate masculinity (−M) und femininity (−F) scales. For gender-related instruments higher scores indicate higher masculinity on masculinity scales, higher femininity on femininity scales and TMF, and higher gender-conformity on CGRB. For criterion characteristics higher scores indicate more frequent contact to lesbians/gay men and straight wo/men, higher voice pitch characteristics, and higher perceived straightness*.

The more gender-conform women and men rated themselves on the TMF, the more likely they were perceived as straight based on voices, faces, and the combination of both (|*r|* > 0.31) however, the correlation for perceived straightness based on voice for women was only by trend). In contrast to men, all voice pitch characteristics correlated significantly with the TMF for women (*r* > 0.32). All correlations were in the expected direction: The higher women spoke on average and the higher their voice pitch range and variability, the more likely they rated themselves as feminine. In contrast, one contact measure showed a significant correlation for men but not for women: The less contact men reported to gay men, the more masculine they rated themselves on the TMF (*r* = −0.35).

The TMF showed 9 out of 16 possible significant correlations which is more than any other gender-related scale. CGRB followed with 6 out of 16 possible significant correlations. Hence, the TMF showed higher convergent validity than the other gender-related scales.

#### Test-retest reliability and predictive validity

Table 11 contains findings regarding test-retest reliability and predictive validity. According to the intercorrelation of TMF scores at first and second measurement, 1-year reliability for the TMF was 0.75 and higher than for the GEPAQ-F, though inter-correlations for the GEPAQ-M were even higher than for the TMF. Hypothesis 12 was confirmed.

**Table 11:** **Reliabilities and correlations for gender-related measures between first (columns) and second (rows) Measurement in Study 3**.

	**Alpha**	**TMF^a^**	**GEPAQ-M^a^**	**GEPAQ-F^a^**
TMF^b^	0.87	0.75	(−0.08)	0.49
GEPAQ-M^b^	0.73	−0.32	0.89	(−0.25)
GEPAQ-F^b^	0.75	0.35	(0.03)	0.65

*Internal consistencies for second measurement are presented in the first column. Test-retest reliabilities are presented on the diagonal. All correlations are statistically significant at α ≤ 0.05 except for those in parentheses. Abbreviations: TMF, Traditional Masculinity-Femininity; GEPAQ, German Extended Personality Attributes Questionnaire. Endings indicate masculinity (−M) und femininity (−F) scales. Higher scores indicate higher masculinity on masculinity scales and higher femininity on femininity scales and TMF*.

a *First measurement*.

b *Second measurement*.

In order to test its predictive value, the TMF at the first measurement was correlated with GEPAQ-M and GEPAQ-F at the second measurement. As can be seen in Table 11, both correlations were significant, of moderate size, and in the expected directions, confirming Hypothesis 13.

### Summary of findings

In Study 2, we found that all TMF items loaded strongly on one single factor at first and second measurement, replicating the pilot study and confirming Hypothesis 1 again. The TMF showed sufficient reliabilities for women and men. Confirming Hypotheses 2, 3, and 4, the TMF turned out to be the best gender-related instrument for differentiating straight and gay men at first and second measurement and lesbians and straight women compared to all other scales used in Study 2 (see Table 4). In line with gender self-stereotyping and contradicting implicit gender inversion theory, gay men showed lower femininity/higher masculinity than lesbians. The evidence for high incremental validity in predicting women's sexual orientation from Study 1 could not be replicated nor extended to men.

Whereas, lesbians and straight women differed descriptively, but not significantly in GRB-M (see Table 4), in the logistic regression analysis (see Table 7), GRB-M predicted women's sexual orientation in a significant way in Step 2, along with GRB-F. We assume that the inclusion of GRB-F in the regression model reduced apparent error variance and thus changed the relation between GRB-M and sexual orientation from descriptive to statistically significant. However, as GRB-M was again non-significant in Step 3 of the regression model, we suggest that masculine everyday behavior was not strongly related to sexual orientation in our women's sample. However, when including adjective-based instruments only, TMF predicted sexual orientation in women and men better than established adjective -based instruments.

Partially confirming Hypothesis 7, the TMF showed moderate correlations with some other gender-related scales. Importantly, the TMF was connected to multiple criterion characteristics for women (e.g., higher femininity was accompanied by more gender-conform voice pitch characteristics) and men (e.g., higher masculinity was associated with less frequent contact to gay men) and outperformed other gender-related scales.

The TMF revealed moderate test-retest-reliability and predictive validity confirming Hypotheses 12 and 13. Scores on the first TMF measurement predicted scores on GEPAQ-M and GEPAQ-F at second measurement.

## General discussion

Gender research has developed many instruments to measure different aspects of self-ascriptions of gender stereotypical features, including attributes, behaviors, interests, and attitudes (Beere, 1990). Supplementing these scales, the TMF scale is designed as an instrument for globally assessing people's overall, or “core,” masculinity-femininity. The TMF was shown to reliably measure an underlying, one-dimensional construct, and it was found to be a valid instrument for assessing masculinity-femininity because it (a) successfully differentiated between groups that were expected to differ (women vs. men, lesbians/gay men vs. straight women and men) and (b) it correlated moderately with other gender-related instruments, such as the Bem Sex Role Inventory (BSRI; Bem, 1974) and the German Extended Personal Attributes Questionnaire (GEPAQ; Runge et al., 1981). Whereas, some well-established, adjective-based scales (e.g., BSRI, GEPAQ) have shown shortcomings in differentiating women and men in recent years (Sczesny et al., 2004; Evers and Sieverding, 2014), our findings of consistent group differences support the TMF as a new tool for measuring gender-role self-concept.

### Dimensionality of the TMF

In line with Choi and Fuqua (2003), high correlations between the separate TMF femininity and masculinity scales as shown in Study 1 suggest a bipolar, one-dimensional use of this instrument reflecting laypersons' ideas of masculinity and femininity as two extremes of one continuum. This is also in line with findings reported by Spence and Bruckner (2000, see also Sánchez and Vilain, 2012). All items were shown to load on one factor and represent a one-dimensional construct (masculinity-femininity). This finding should be not taken as hint that one-dimensional masculinity/femininity models generally outperform two dimensional ones (e.g., agency, communion; competence, warmth; instrumentality, expressivity), but that all TMF items appear to refer to the same underlying construct. Moreover, in spite of its brevity, the TMF showed high internal consistencies across all studies as well as satisfactory test-retest reliability (in a sample of men). However, the one-dimensionality of the TMF was demonstrated with participants identifying themselves as women or men. Possibly, the two-dimensional TMF version is superior than the one-dimensional version for samples that comprise a larger number of participants transgressing or rejecting the binary gender system (e.g., transgender and queer people). Future research is needed to clarify that question.

One could object against using the bipolar TMF scale that its midpoint is ambiguous. In other words: what does a score of “4” mean? One could imagine that people scoring either high or low on both dimensions would erroneously be treated as one group. However, according to the high correlations between the separate TMF masculinity and femininity scales (Study 1) and a supplementary graphical scatterplot analysis we did, we found no groups of high/high (i.e., androgyny) or low/low scorers (i.e., undifferentiated). Hence, it can be deduced that people in our samples who scored close to “4” believed themselves to be moderately feminine and masculine.

### Contextualizing validity findings

In terms of validity, using a known-groups approach as an established psychological method for validity tests (e.g., Howitt and Cramer, 2008), the TMF repeatedly showed expected gender differences, with men scoring higher on masculinity and lower on femininity than women. With reference to sexual orientation, straight and bisexual women rated themselves higher on femininity and lower on masculinity than lesbians did (Study 1). Moreover, the TMF was the only gender-related scale used in the present study that distinguished straight men, gay men, lesbians, and straight women (from high masculinity/low femininity to low masculinity/high femininity, Study 2) which supports gender self-stereotyping rather than implicit gender inversion theory (Kite and Deaux, 1987). According to implicit gender inversion theory, gay men should have scored higher than lesbians on femininity and lower on masculinity, which was not the case in our sample. It appears that gay men and lesbians rather self-stereotype as men and women, respectively, and thus construct their self-concept in line with their gender group. Based on these findings, we conclude that the TMF's ability for determining gender and sexual orientation was generally high, and higher than that of all other gender-related measures investigated in the present studies. Finally, we found evidence for the idea that differences in “core” masculinity and femininity measured by the TMF underlie differences in lesbians' and gay men's vs. straight women and men's self-ascriptions in gender typicality measured by other scales, such as the BSRI (see Study 1). Hence, the TMF was shown to be a valid scale for assessing gender-role self-concept.

It was expected that the TMF would correlate moderately with other gender-related scales. That was the case for all gender-related scales in Study 1 where only a female sample was tested. This indicates that the TMF measures other aspects of people's conceptualizations of their own masculinity/femininity than the BSRI or the Gender-Role Behavior Scale (Athenstaedt, 2003) and complements them well. An explanation for this findings is that the TMF does not measure attributes associated with masculinity/femininity, but rather, these constructs themselves. Only correlations with the Childhood Gender-Behavior Scale were high, which could be due to selective memory recall and hence reflect current gender-related self-assessment (see Bailey and Zucker, 1995) measured with the TMF. Alternatively, the high correlation is due to actual gender differences during childhood, which would be a hint for constancy of conceptualizations of people's own masculinity/femininity. Correlations between the TMF and gender-related scales were smaller for a second sample of women (Study 2) which could be due to differences in sampling and substitutions of scales (e.g., instead of the Bem Sex Role Inventory, the Personal Attributes Questionnaire was used). Connected to that, the incremental validity of the TMF for predicting women's sexual orientation was demonstrated in Study 1 only. However, the male sample in Study 2 showed overall moderate correlations of the TMF and gender-related scales, but no additional ability of the TMF to predict sexual orientation. The fact that the TMF did not always demonstrate additional predictive value for explaining differences between groups does not indicate that it is superfluous. Rather, other facets of self-ascribed masculinity/femininity, such as everyday behavior, turned out to be highly capable of predicting sexual orientation as well. And the TMF predicted sexual orientation still better than established adjective-based instruments in women and men in Study 2 (which was demonstrated after excluding the most predictive scales).

To deal with a common critique that self-report instruments measure differences in social desirability rather than true differences, we used an implicit measure of women's self-feminine vs. self-masculine associations. Study 1 showed that the correlations of these associations were higher for the TMF than for self-ratings of traits or behaviors. This is a strong hint that the TMF is able to reflect “true” differences in core masculinity/femininity rather than social desirability only. It is also a substantive finding of the present studies that goes beyond mere scale validation.

In a similar vein, in order to test the criterion validity of the TMF, we selected several criterion characteristics which can be categorized into three groups (Study 2): These included contact to same-gender straight women/men and lesbians/gay men, voice pitch features, and assessment of sexual orientation by laypersons based on visual and auditory stimuli. Correlation analyses showed that gender-conformity on the TMF was significantly linked to perceived straightness for almost each presentation mode (voice, face, and the combination of both) for men and women. Moreover, higher femininity in women was associated with higher voice pitch features (average, variability, and range) and higher masculinity in men was connected to less contact to gay men. Compared to other gender-related scales, the TMF was superior in convergent validity. Taken together, self-ratings of masculinity/femininity go along not only with gender and sexual orientation differences, but also with differences in social behavior (i.e., contact to same-gender people differing in sexual orientation), with objective voice characteristics, and with assessments of sexual orientation based on facial and voice features. In sum, this indicates that the TMF measures something fundamental regarding gender-related self-assessment. It is also another substantive finding of the present studies that goes beyond mere scale validation. A limitation is that patterns of findings partially differed between women and men, and which specific criteria mattered in which sub-sample appeared a bit arbitrary (e.g., voice pitch features for women and contact variables for men). It appears that women and men express their masculinity/femininity in different ways, which is an interesting topic for future research.

### Theoretical considerations regarding the TMF

One might assume that a one-item-measure could be sufficient for assessing masculinity/femininity by simply asking how masculine/feminine people believe themselves to be. We checked this idea in an exemplary fashion for Study 2 using the “I consider myself as…”-item for a comparative analysis because of highest corrected item-total correlations for the whole sample in the Pilot Study. However, in every case (determining and predicting gender and sexual orientation, convergent, and criterion validity), as a rule the TMF was better than the one-item-measure (e.g., compared to the one-item measure the TMF showed higher correlations for almost all gender-related measures in the male subsample except for GEPAQ-M where a higher correlation was found for the one-item measure). This is in line with state-of-the-art conceptions in psychological assessment that consider every item in a scale to be a piece of puzzle and hence uncover a different detail of a somewhat bigger picture (Bühner, 2010). Moreover, it is also consistent with Constantinople's (1973) view that the masculinity/femininity-construct is captured best when gender role adoption, preference, and identity are measured in conjunction.

The TMF is designed as a self-assessment instrument for masculinity-femininity on a rather global level with regard to two different respects. First, the TMF is based on a trait rather than a normative approach (see Thompson and Bennet, 2015) and conceptualizes masculinity-femininity as a long-term characteristic varying between people. However, it does not exclude variation on masculinity-femininity within a person depending on different social, temporal, or regional contexts. Its focus is on a trait-like (global) average score across contexts. Second, it is more global because it focuses on a higher-order masculinity-femininity construct which is beyond specific components such as traits, interests, physical characteristics, or attitudes, and asks for an aggregated self-assessment across these domains. The high test-retest reliability obtained over a 1-year period indicated stability rather than variance. However, it would be interesting to know which components mainly account for an individual's judgment of their own gender-related identity. The TMF could be a valuable instrument for future research dealing with that question.

In spite of this trait-like approach, the TMF is based on the idea that masculinity/femininity is socially determined (see Smiler, 2004). The scale is about how people relate or conform to social standards (how masculine/feminine do they believe themselves to be?), but not how they consider social norms to be appropriate for men and women (i.e., what people consider as masculine/feminine). To trigger a reference to social norms in the participants' minds when testing gender-role identity aspects, we used the term “traditionally” in the beginning of the corresponding items. However, the TMF does not measure if participants' conceptions of gender-role identity aspects correspond to traditional views. Thus, we concede that there could be variations in people's understanding of “traditionally” which could affect their self-evaluations. However, large differences are not likely because people within one culture know about traditional gender roles.

Because of the TMF's broader scope compared to established scales, such as the BSRI and PAQ, it is reasonable to be positive about the TMF's ability of measuring masculinity/femininity also in the future. Hence, it seems plausible that the problem of item aging is mitigated for the near future because of the more global wordings. Additionally, we are positive that the TMF can be used in different countries and cultures because of its global level of measurement. To date, the TMF has only been applied to one other German sample by Roth and Mazziotta (2015). They found that the TMF was moderately connected to different aspects of social identification with one's own gender in the expected directions for men and women. According to Leach et al. (2008), social identification is a multidimensional multicomponent higher order construct. The TMF was shown to be linked to almost all of its different components (individual self-stereotyping, in-group homogeneity, satisfaction, solidarity, and centrality) for women and men except for in-group homogeneity for men. Future research should provide evidence for the applicability in non-German samples.

### Concluding remarks

In a nutshell, as long as societies assume differences in interests, attitudes, clothing style, and behavior between women and men, we suggest that the TMF provides a valuable addition to researchers' toolbox. For example, are self-ratings on the TMF related to biological markers of masculinity-femininity such as waist-to-hip ratio and finger length (i.e., two-digit-four-digit ratio)? Do self-ratings on the TMF predict behaviors in which large gender differences have been observed, such as socio-sexuality or animal cruelty? Are self-ratings on the TMF related to performance in domains where gender differences are reliable, such as mental rotation? Finally, are self-ratings on the TMF related to personality traits in which gender differences have been observed, such as self-esteem and social dominance orientation? Generally, we believe that many different research questions related to gender-related self-assessments could benefit from using the TMF.

## Author contributions

Substantial contributions to the conception or design of the work and the acquisition and analysis of the data: SK, MS; interpretation of data for the work: SK, MS, CN. Drafting the work or revising it critically for important intellectual content: SK, MS, CN. Final approval of the version to be published: SK, MS, CN. Agreement to be accountable for all aspects of the work in ensuring that questions related to the accuracy or integrity of any part of the work are appropriately investigated and resolved: SK, MS. We thank Kornelia Schertzl, Karoline Nestler, Dirk Hertrampf, Felicia Schuld, and Alexander Makosch for help with data collection, Susanne Fuchs, Stefanie Jannedy, and Joerg Dreyer for providing laboratories in the Zentrum fuer Allgemeine Sprachwissenschaft, Berlin, and Anders Sonderlund for language editing. Additionally, we thank Julia Scholz and the reviewers for critical and valuable comments on earlier versions of the manuscript. The TMF was originally developed by MS and Kornelia Schertzl.

## Funding

The current research was partially funded by grants from the German Research Foundation (DFG, STE 938/10-2, FOR 1097, and STE 938/11-1).

### Conflict of interest statement

The authors declare that the research was conducted in the absence of any commercial or financial relationships that could be construed as a potential conflict of interest.

## Abbreviations

BSRI: Bem Sex Role Inventory
BSRI-F: Bem Sex Role Inventory-femininity scale
BSRI-M: Bem Sex Role Inventory-masculinity scale
CGRB: Childhood Gender Role Behavior
f0: fundamental frequency
GEPAQ: German Extended Personal Attributes Questionnaire
GEPAQ-F: German Extended Personal Attributes Questionnaire-femininity scale
GEPAQ-M: German Extended Personal Attributes Questionnaire-masculinity scale
GRB: Gender Role Behavior
GRB-F: Gender Role Behavior-femininity scale
GRB-M: Gender Role Behavior-masculinity scale
IAT: Implicit Association Test
PAQ: Personal Attributes Questionnaire
TMF: Traditional Masculinity-Femininity
TMF-F: Traditional Masculinity-Femininity-femininity scale
TMF-M: Traditional Masculinity-Femininity-masculinity scale.
