# Supplementary material for: Traditional Masculinity and Femininity: Validation of a New Scale Assessing Gender Roles
Source: Front Psychol. 2016 Jul 5;7:956. doi: 10.3389/fpsyg.2016.00956 (PMC4932111; doi:10.3389/fpsyg.2016.00956)
Supplement: Supplementary file 1 [file DataSheet1.docx]

Appendix A

**A1. The Traditional Masculinity-Femininity Scale (TMF) in English translation [with original German wording]**

I consider myself as…

[Ich empfinde mich selbst als…]

Ideally, I would like to be…

[Idealerweise wäre ich gern… ]

Traditionally, my interests would be considered as…

[Traditionellerweise würden meine Interessen angesehen werden als…]

Traditionally, my attitudes and beliefs would be considered as…

[Traditionellerweise würden meine Einstellungen und Ansichten angesehen werden als…]

Traditionally, my behavior would be considered as…

[Traditionellerweise würde mein Verhalten angesehen werden als…]

Traditionally, my outer appearance would be considered as…

[Traditionellerweise würde meine äußere Erscheinung angesehen werden als…]

Scales ranged from 1 (*not at all masculine*) to 7 (*totally masculine*) and from 1 (*not at all feminine*) to 7 (*totally feminine*) in Study 1 and from 1 (*totally masculine*) to 7 (*totally feminine*) in the pilot study and in Studies 2-3.

**A2. Childhood Gender-Role Behavior Scale (CGRB)**

1. In my childhood I liked being a girl.
2. In my childhood I would rather have been a boy.
3. In my childhood I behaved like a “typical girl”.
4. In my childhood I behaved like a “typical boy”.
5. In my childhood I played typical girl games.
6. In my childhood I played typical boy games.
7. In my childhood I played with girls.
8. In my childhood I played with boys.
9. In my childhood I wanted to wear dresses and skirts.
10. In my childhood I wanted to wear trousers.

Scales ranged from 1 (*never applied*) to 7 (*always applied*) in Study 1 and from 1 (*I strongly disagree*) to 5 (*I strongly agree*) in Study 2.

Appendix B

**Table 1** Factor, pattern, and structure matrix of the exploratory factor analysis with a two-factor solution and the confirmatory one-factor analysis of the TMF in Study 1.

|  | Factor Matrix | |  | Pattern Matrix | |  | Structure Matrix | |  | Factor matrix CFA |
| --- | --- | --- | --- | --- | --- | --- | --- | --- | --- | --- |
|  | F1 | F2 |  | F1 | F2 |  | F1 | F2 |  |  |
| - 1. (F) I consider myself as… | .71 | .39 |  | -.09 | .87 |  | .51 | .81 |  | .70 |
| 1. (F) Ideally, I would like to be... | .77 | .43 |  | -.11 | .95 |  | .55 | .88 |  | .75 |
| 1. (F) Traditionally, my interests would be considered as… | .76 | -.27 |  | .76 | .06 |  | .80 | .58 |  | .75 |
| 1. (F) Traditionally, my attitudes and beliefs would be considered as… | .78 | -.37 |  | .90 | -.06 |  | .86 | .56 |  | .77 |
| 1. (F) Traditionally, my behavior would be considered as… | .87 | -.22 |  | .77 | .18 |  | .89 | .70 |  | .87 |
| 1. (F) Traditionally, my outer appearance would be considered as… | .77 | .27 |  | .10 | .74 |  | .61 | .81 |  | .77 |
| 1. (M) I consider myself as… | -.80 | -.21 |  | -.19 | -.69 |  | -.66 | -.82 |  | -.80 |
| 1. (M) Ideally, I would like to be... | -.67 | -.23 |  | -.09 | -.64 |  | -.53 | -.70 |  | -.67 |
| 1. (M) Traditionally, my interests would be considered as… | -.81 | .27 |  | -.81 | -.07 |  | -.85 | -.62 |  | -.81 |
| 1. (M) Traditionally, my attitudes and beliefs would be considered as… | -.69 | .36 |  | -.84 | .10 |  | -.76 | -.48 |  | -.68 |
| 1. (M) Traditionally, my behavior would be considered as… | -.79 | .22 |  | -.73 | -.13 |  | -.82 | -.63 |  | -.79 |
| 1. (M) Traditionally, my outer appearance would be considered as… | -.75 | -.25 |  | -.11 | -.71 |  | -.60 | -.78 |  | -.75 |

*Note.* Scale ranged from 1 - “not all masculine/feminine” to 7 - “very masculine/feminine”. Abbreviations: Factor 1 (F1), Factor 2 (F2), confirmatory factor analysis (CFA), femininity scale (F), masculinity scale (M)
